# Supplementary material for: Quasi-2D Fermi surface in the anomalous superconductor UTe2
Source: Nat Commun. 2024 Jan 3;15:223. doi: 10.1038/s41467-023-44110-4 (PMC10764345; doi:10.1038/s41467-023-44110-4)
Supplement: Supplementary file 1 — Supplementary information [file 41467_2023_44110_MOESM1_ESM.pdf]

# Supplementary information for:

## Quasi-2D Fermi surface in the anomalous superconductor $UTe_2$

A. G. Eaton, T. I. Weinberger, N. J. M. Popiel, Z. Wu, A. J. Hickey, A. Cabala, J. Pospíšil, J. Prokleška, T. Haidamak, G. Bastien, P. Opletal, H. Sakai, Y. Haga, R. Nowell, S. M. Benjamin, V. Sechovský, G. G. Lonzarich, F. M. Grosche, and M. Vališka.

Correspondence to: alex.eaton@phy.cam.ac.uk

## Poor correspondence between dHvA effect data and DFT-calculated Fermi surfaces

We performed density functional theory (DFT) calculations for $UTe_2$ using the full electron, linearised augmented plane-wave package Wien2K.[1] Electronic structures were converged on a $17 \times 17 \times 17$ Monkhorst-Pack $k$-mesh within the Brillouin Zone (BZ) of the primitive unit cell using the Generalised Gradient Approximation (GGA) exchange-correlation potential. A variable Hubbard parameter (U) was utilised, while the static magnetic moment on the uranium ions was constrained to zero. The effects of spin-orbit coupling (SOC) were taken into account. Lattice parameters and internal positions were chosen according to those used in previous DFT attempts at fitting dHvA oscillations in $UTe_2$,[2,3] listed in Table S1. Subsequently, non-self-consistent calculations were performed on a $46 \times 46 \times 46$ $k$-mesh (12996 $k$-points in the irreducible BZ) to obtain a high resolution Fermi surface for further study.

DFT+U+SOC results were consistent with previous work.[3,4] When correlations are neglected, the DFT produces an insulating ground state in $UTe_2$. However, applying a moderate repulsive potential to the U-5$f$ electrons through a Hubbard U induces an insulator to metal transition at $\sim$ 1 eV.[4] The geometry and topology of the computed Fermi surfaces was exam-

| Species | *x* | *y* | *z* |
|---|---|---|---|
| U | 0 | 0 | 0.13480 |
| Te1 | 0.5 | 0 | 0.29770 |
| Te2 | 0 | 0.2510 | 0.5 |

**Table S1: The internal atomic positions used in our DFT study of $UTe_2$.** The lattice constants chosen for the calculations were $a$ = 4.1617 Å, $b$ = 6.1276 Å, and $c$ = 13.9650 Å.

ined for U = 1-16 eV. For values of U below 1.7 eV a 3D, toroidal, electron-like contribution to the Fermi surface is recovered, while the hole-like surface consists of a heavily warped cylinder. Above 1.7 eV the toroidal Fermi surface splits into another cylinder whereas the hole-like sheet becomes less warped, resulting in a quasi-2D Fermi surface consisting of two cylinders centred at the **X** and **Y** high-symmetry points.

Here, we plot characteristic Fermi surface calculations for a range of U values from U = 1 eV up to U = 16 eV. We compare the expected angular evolution of the dHvA effect of these calculated Fermi surfaces with the measurements performed in this study and reported in ref.[3] It is clear that none of these calculated Fermi surfaces fully account for the observed dHvA frequency evolution. Namely, none of them are able to account for the existence of a low frequency branch. Further, all the DFT calculations exhibit moderate degrees of corrugative warping resulting in Fermi surface sheets that will produce multiple frequencies along the c-axis, in contradiction to what we have observed (Supplementary Figure S7). Hence, this motivated us to perform Fermi surface simulations guided by the dHvA data, as detailed below.

Visualisation of DFT-generated Fermi surfaces was performed using PyVista-based scripts[5,6] and the corresponding quantum oscillation frequencies were extracted using the SKEAF extremal area program.[7]

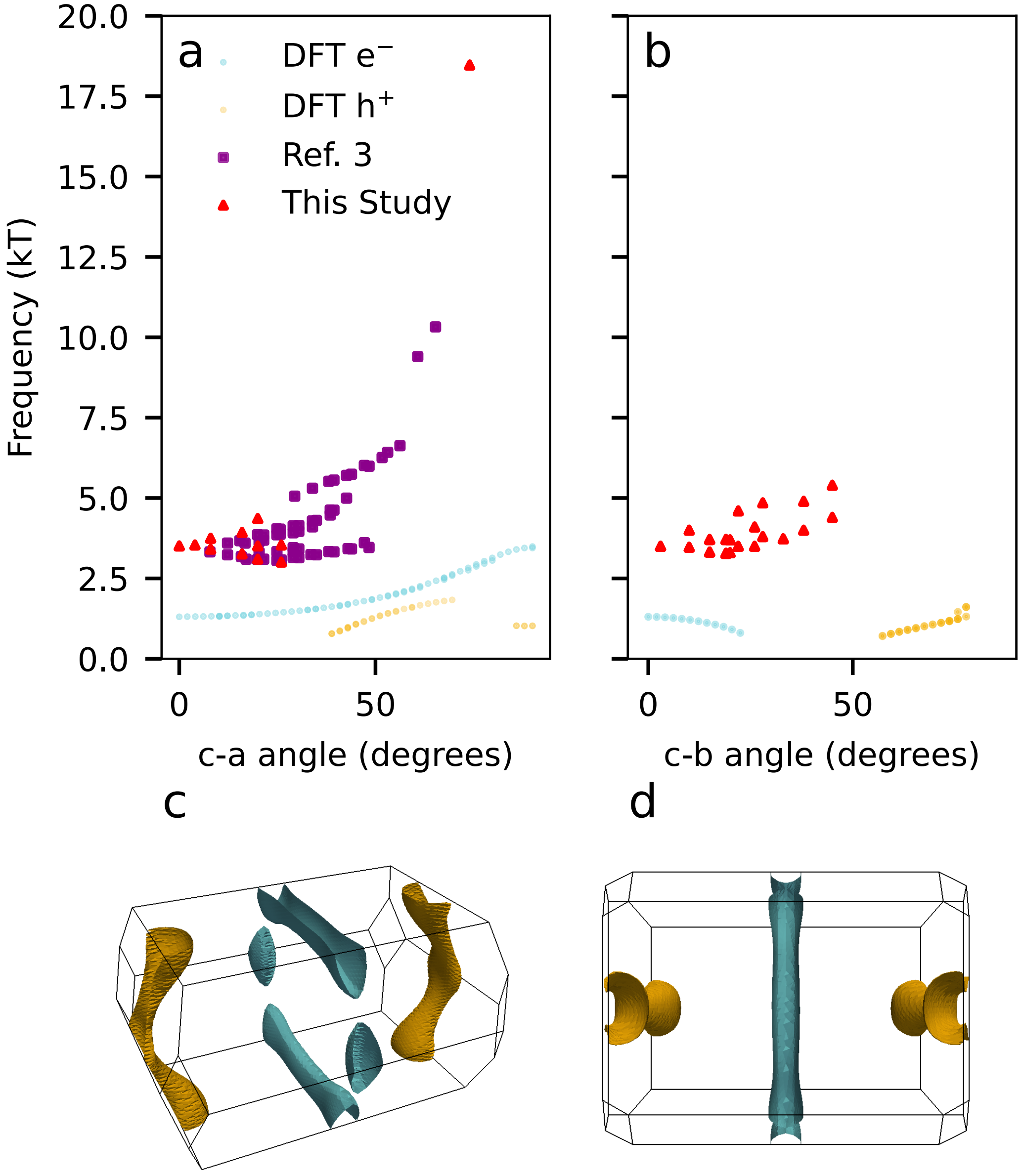


**Fig. S1. The simulated quantum oscillation frequencies (a,b) from the DFT calculated Fermi surface with U = 1.0 eV (c,d).** Low quantum oscillatory frequencies are expected, very different from those observed by experiment.

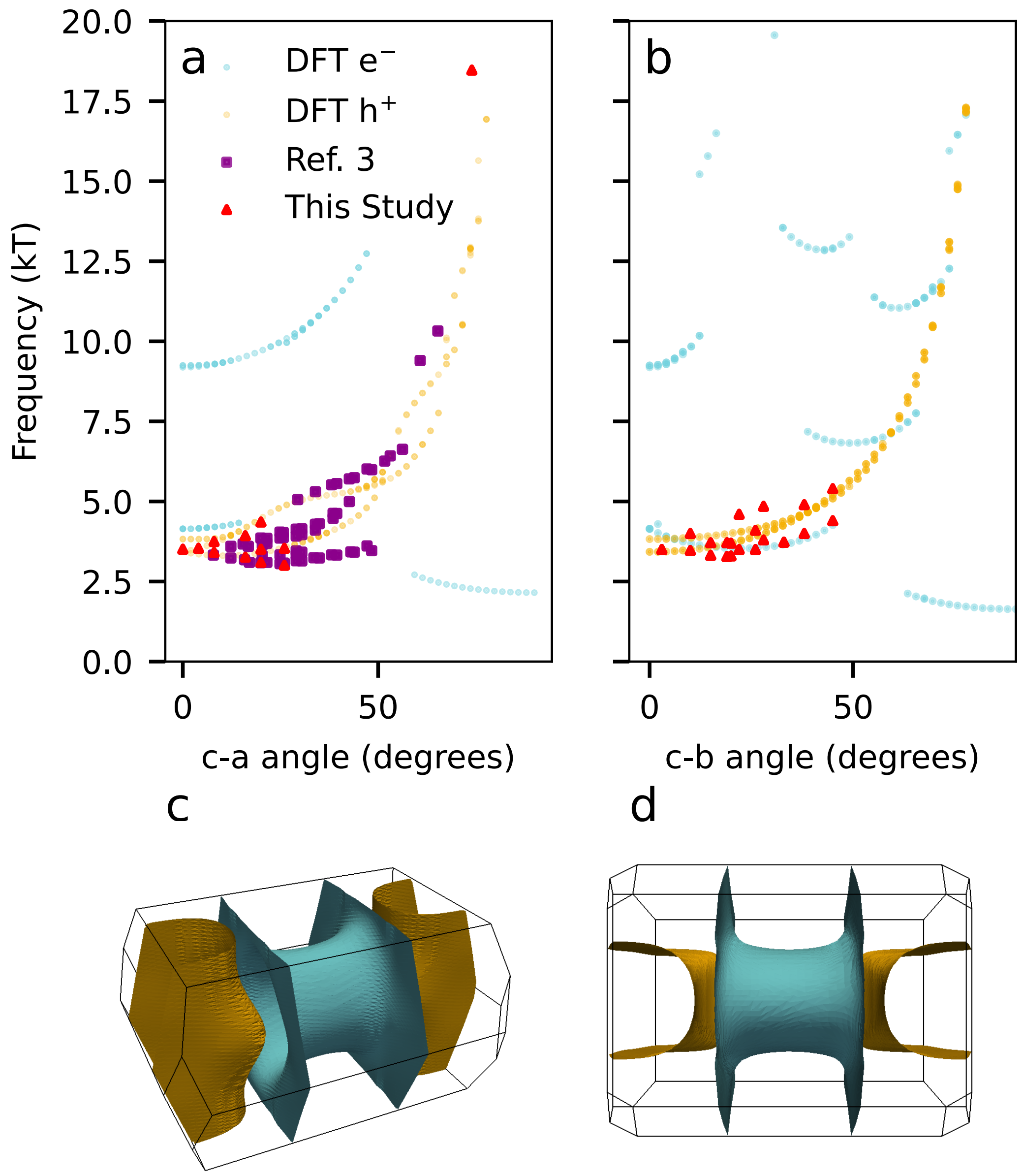


**Fig. S2. The simulated quantum oscillation frequencies (a,b) from the DFT calculated Fermi surface with U = 1.5 eV (c,d).** Multiple frequency branches are expected, in both rotation planes, that are not observed by experiment.

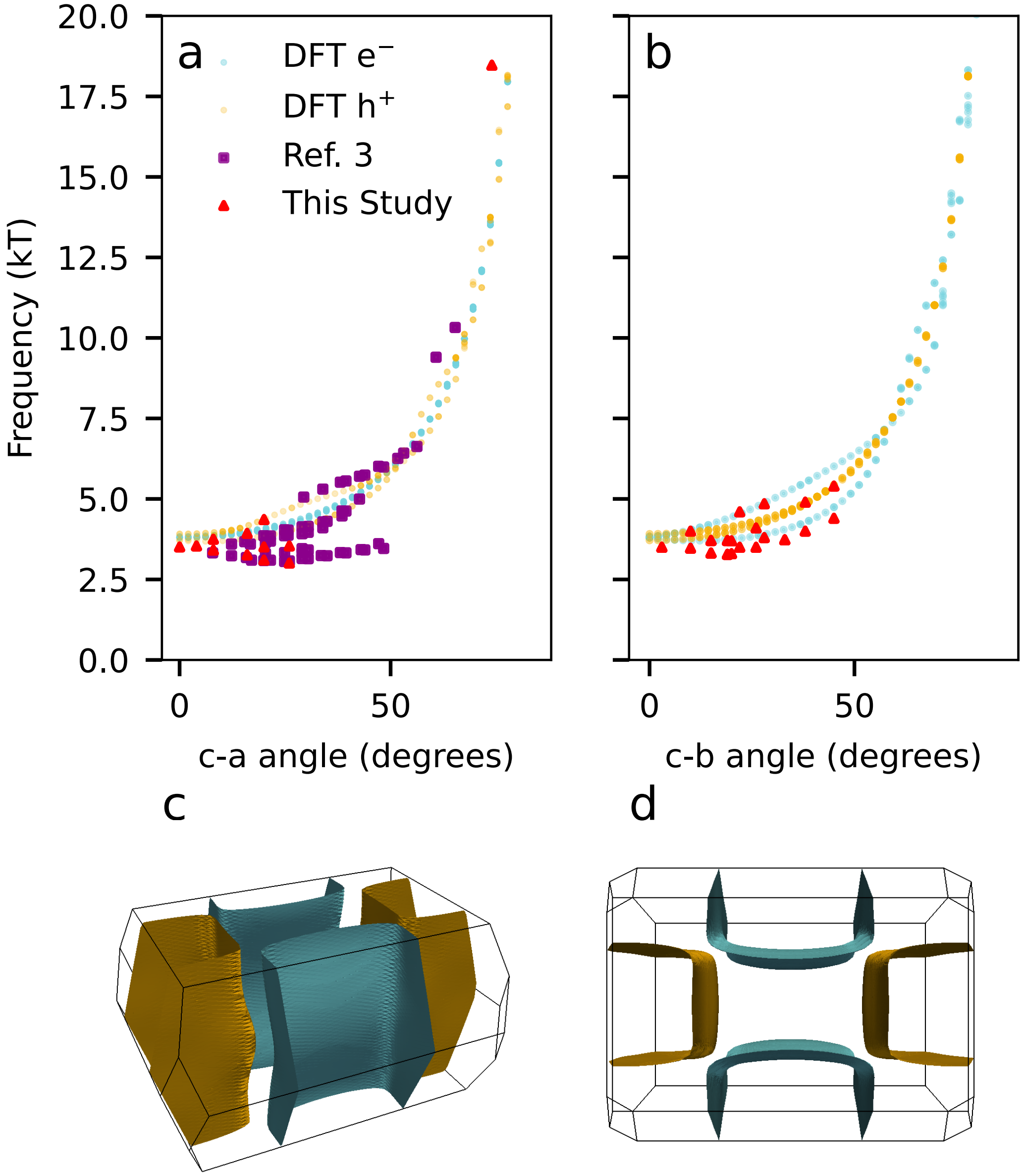


**Fig. S3. The simulated quantum oscillation frequencies (a,b) from the DFT calculated Fermi surface with U = 2.0 eV (c,d).** A large proportion of the angular profile in the $c$-$b$ plane is captured by this Fermi surface calculation. However, in the $c$-$a$ plane the low, spectrally dominant frequency branch is not accounted for.

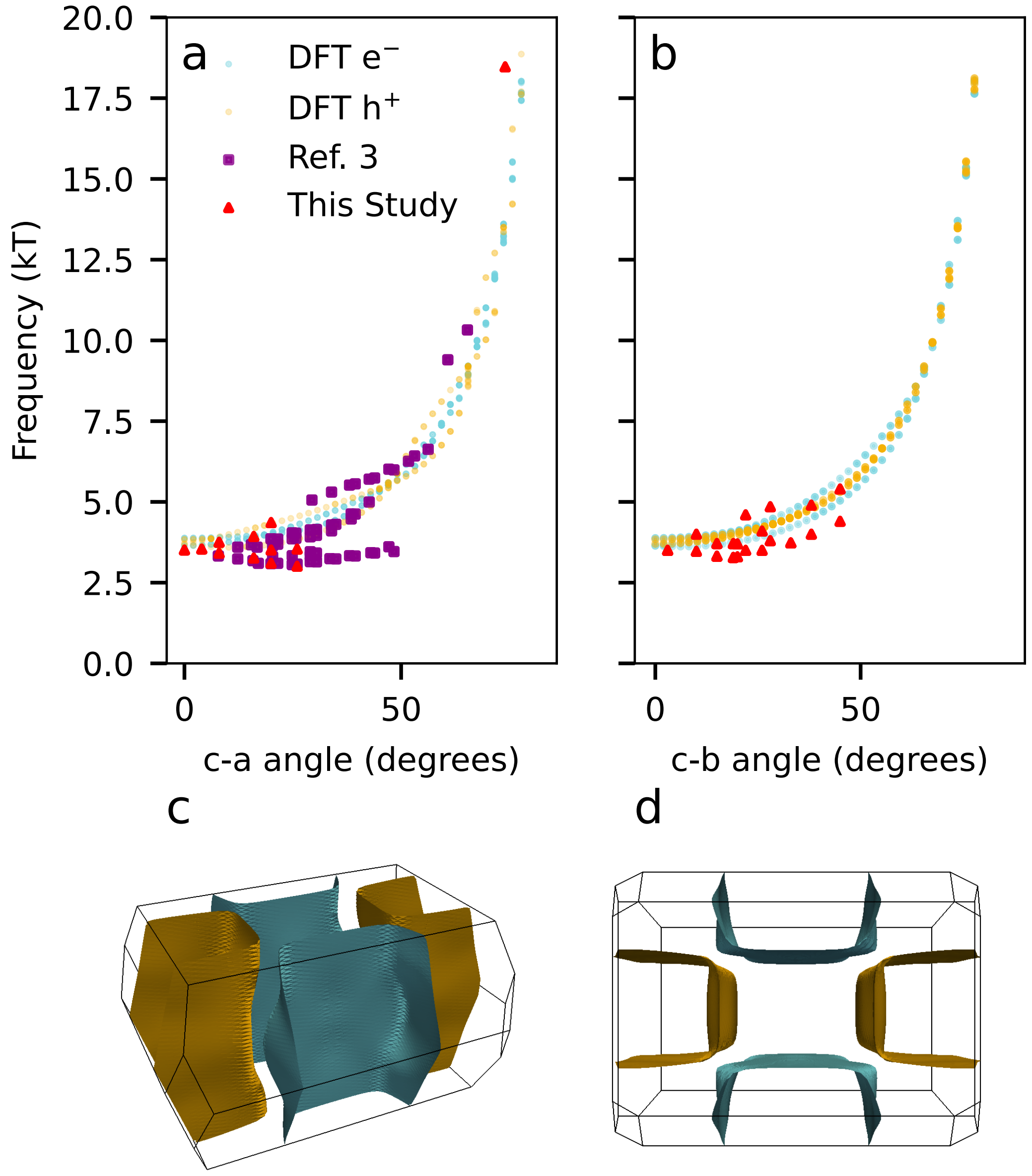


**Fig. S4. The simulated quantum oscillation frequencies (a,b) from the DFT calculated Fermi surface with U = 8.0 eV (c,d).** Again the low, spectrally dominant frequency branch in the $c$-$a$ plane is not accounted for.

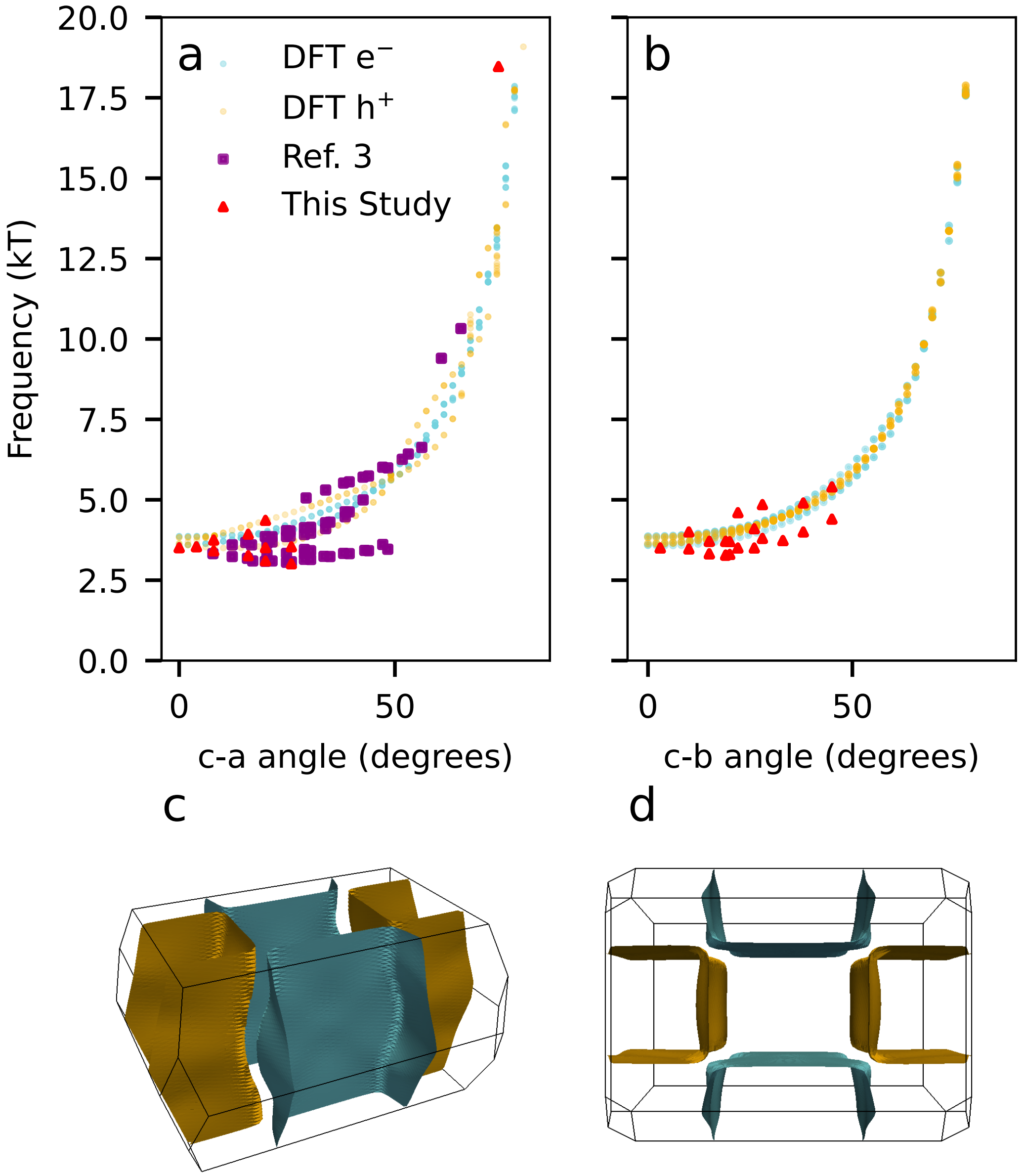


**Fig. S5. The simulated quantum oscillation frequencies (a,b) from the DFT calculated Fermi surface with U = 12.0 eV (c,d).** Again the low, spectrally dominant frequency branch in the $c$-$a$ plane is not accounted for.

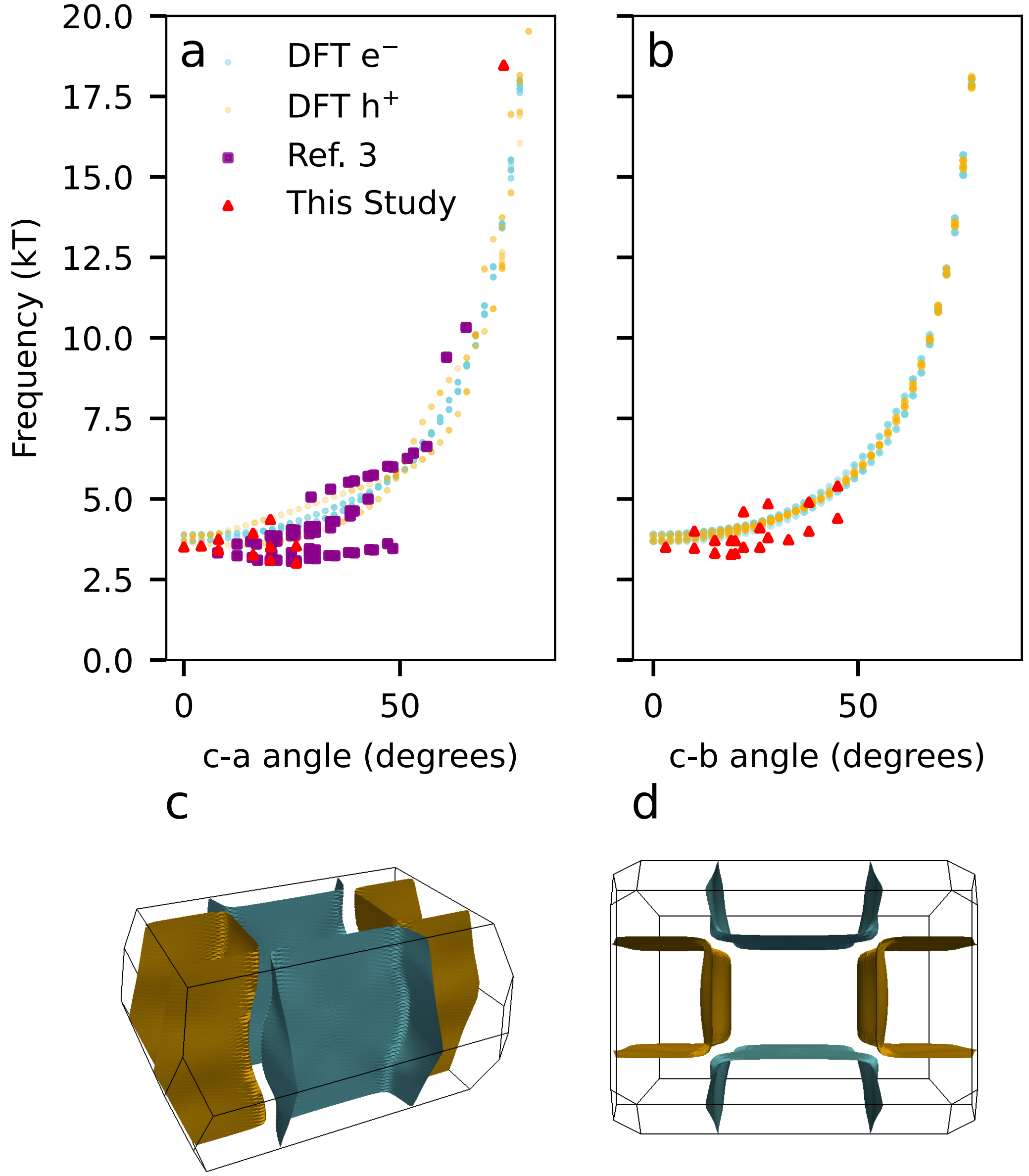


**Fig. S6. The simulated quantum oscillation frequencies (a,b) from the DFT calculated Fermi surface with U = 16.0 eV (c,d).** Again the low, spectrally dominant frequency branch in the $c$-$a$ plane is not accounted for.

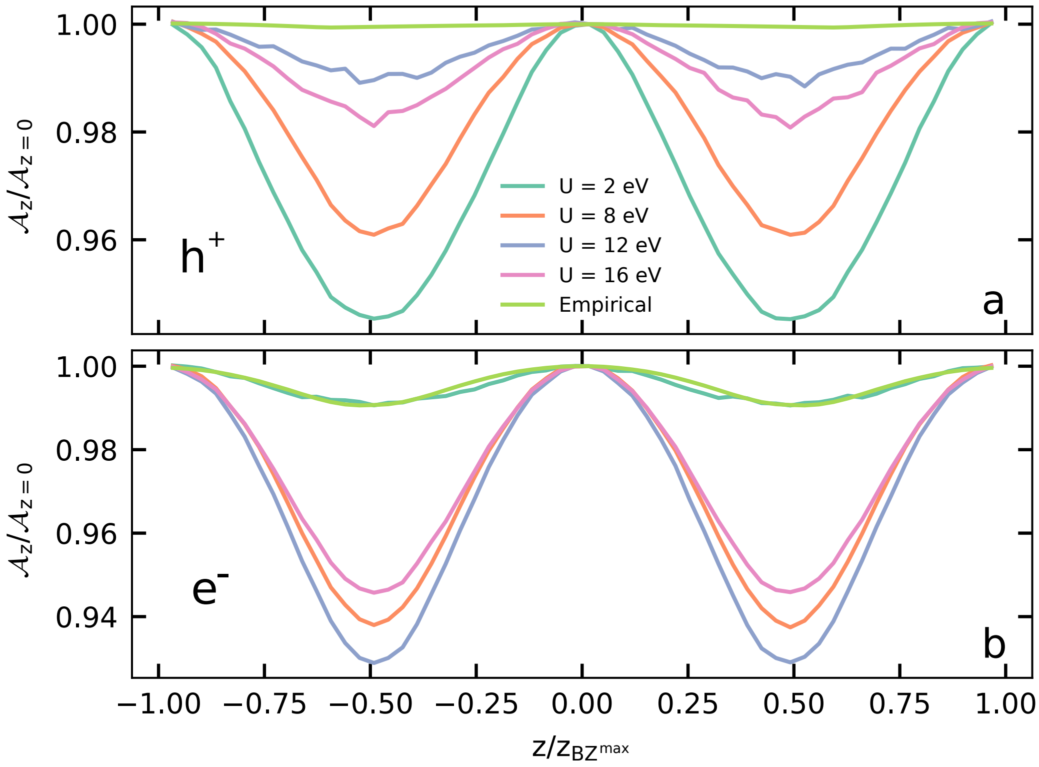


**Fig. S7. Change in Fermi surface cross-sectional area as a function of distance $z$ in the first Brillouin zone**. DFT calculations predict substantially more corrugation than our empirical model which has near-constant cross sectional area for both the hole (a) and electron pockets (b).

## Fermi surface parameterisation

The Fermi surface simulations detailed in the Methods section were generated using a Cartesian reciprocal space basis:

$$\begin{aligned} \vec{e_x} &= 2\pi(1/a, 0, 0), \\ \vec{e_y} &= 2\pi(0, 1/b, 0), \\ \vec{e_z} &= 2\pi(0, 0, 1/c). \end{aligned} \tag{1}$$

This can be related to the actual $k$-space basis as:

$$\begin{aligned} \vec{k_x} &= \vec{e_y} + \vec{e_z} \\ \vec{k_y} &= \vec{e_x} + \vec{e_z} \\ \vec{k_z} &= \vec{e_x} + \vec{e_y}. \end{aligned} \tag{2}$$

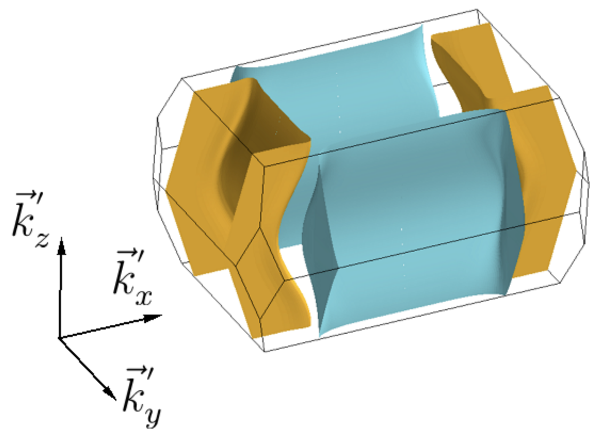


**Fig. S8. The reciprocal space basis used to parameterise the Fermi surface.** A Cartesian coordinate system was used since it has an intuitive relation with the cylindrical polars used to parameterise the Fermi surface.

## Fermi surface simulations

As discussed above, DFT calculations were unable to accurately describe the measured quantum oscillation data. On close inspection, the dHvA effect data exhibit some key features:

1. At 0°, all frequencies collapse onto a single point, indicating that the area of all surfaces when viewed down the $c$-axis must be extremely similar.

2. When initially rotating away from $\vec{c}$ towards $\vec{a}$, the frequencies split into three branches, with one branch that decreases in frequency, which would appear at odds with a cylindrical Fermi surface of circular cross-section.

3. At angles close to 90° the the frequencies go as $\frac{1}{\cos\theta}$ with only one (fast) frequency observable at 74°, indicative of cylindrical Fermi surfaces of similar area again.

Initially, the behaviour of the quantum oscillation data at 0° and high angles seems to contradict the behaviour seen at intermediate angles. However, from the angle dependence of the oscillations and from DFT calculations, several features of the Fermi surface can be deduced. DFT calculations suggest that the Fermi surface does indeed consist of two 'squircular' cylinders. The 0° behaviour indicates that both cylinders must have very similar areas but also that their area cannot be very warped as a function of $k_z$ otherwise this would result in a number of frequencies at 0°. However, the mid-angle frequencies show neck-and-belly behaviour as well as a branch that decreases in frequency, which would indicate warping along $k_z$. To reconcile these behaviours, it is noted that the symmetry of the Brillouin Zone allows spatial warping along $k_z$. What this means is that the Fermi surface consists of cylinders of constant area which follow a sinusoidally oscillating path in the $k_x/k_y$ plane as a function of $k_z$ (Supplementary Figure S9).

In DFT, changing U in the range 2-16 eV modifies the direction and amplitude of the above warping, likely due to changes in the hybridisation of the bands. However, the DFT results could

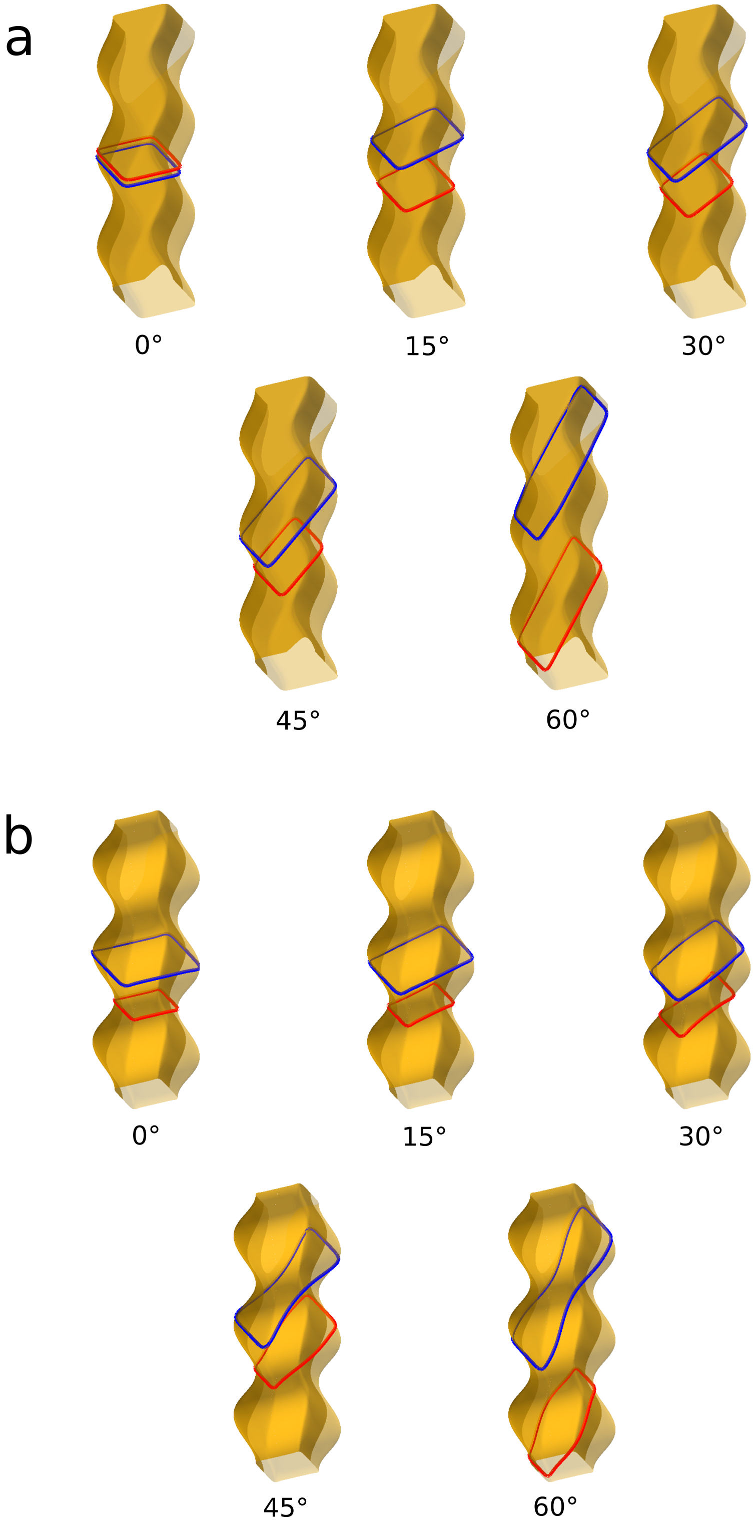


**Fig. S9. Comparison of the maximal (blue) and minimal (red) semi-classical orbits around undulating (a) and corrugated (b) cylindrical Fermi surfaces for different field tilt angles in the $a$-$c$ plane.** We find that an undulating surface allows for degenerate orbits along the $c$-axis that split into an increasing and decreasing branch off-axis. At some critical angle between 45°and 60°the minimal frequency will rapidly increase. This profile is able to describe all the features of the dHvA oscillations. For a corrugated, neck-and-belly type surface, on-axis the orbits are degenerate and off-axis follow a $1/(1-\cos(\theta))$ relation, at odds with measurement.

not completely capture the angular frequency evolution observed in the quantum oscillation data (see Supplementary Figures S1-6).

Instead, an approach similar to the work of Bergemann et al.[8] was adopted. For $UTe_2$, the squared-off shape of the cylinders means that the Fermi surface can be described as a superposition of super-ellipses defined as:

$$\left|\frac{q_x}{R_a}\right|^n + \left|\frac{q_y}{R_b}\right|^n = 1. \tag{3}$$

Therefore, the surface vectors can be defined as

$$\begin{aligned} q_x(t) &= \sum_n A_n |\cos t|^{\frac{2}{n}} \cdot R_a \operatorname{sgn}(\cos t) \\ q_y(t) &= \sum_n A_n |\sin t|^{\frac{2}{n}} \cdot R_b \operatorname{sgn}(\sin t) \end{aligned} \qquad t \in [0, 2\pi] \tag{4}$$

where $R_a$ and $R_b$ are the semi-diameters in the $q_x$ and $q_y$ direction respectively. $q_x$ and $q_y$ are defined according to orthogonal basis vectors, not the reciprocal lattice vectors (see Supplementary Figure S7). The exact shape of the Fermi surface in the $\vec{e}_x - \vec{e}_y$ plane does not have a strong influence on the simulated oscillations and so the combination of super-ellipses was chosen such that it reproduced the in-plane shape of the U = 8 eV DFT calculation. The dominant super-ellipse contribution comes from $n = 5$ (Supplementary Figure S10).

The centres of the super-ellipses trace out a sinusoidal path in reciprocal space that can be parameterised as

$$\begin{aligned} p_x(u) &= w_a \cos(u) \\ p_y(u) &= w_b \cos(u) \qquad u \in [-\pi, \pi] \\ p_z(u) &= u \end{aligned} \tag{5}$$

where $w_a$ and $w_b$ are the warping parameters in the $\vec{e}_x$ and $\vec{e}_y$ directions respectively. Physically, this may correspond to hybridisation between U and Te orbitals governed by the respective

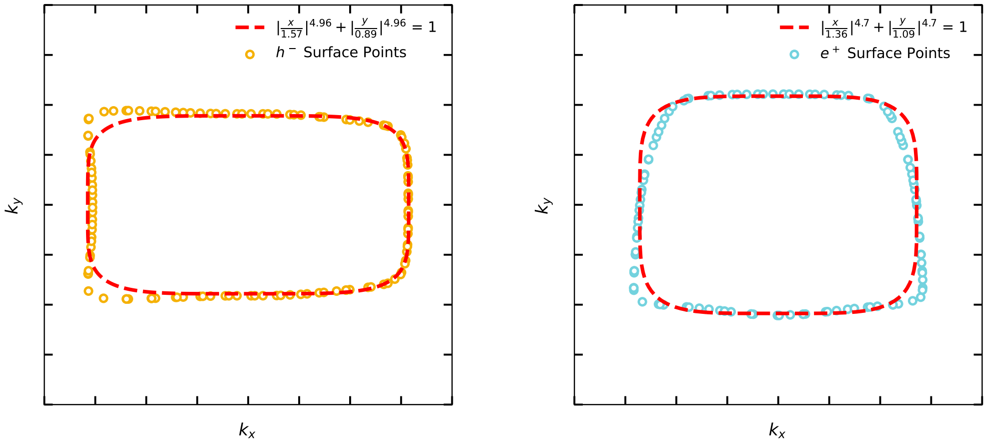


**Fig. S10. Fits for the dominant super-ellipsoidal portions of the electron and hole cylinder from a DFT calculation with U = 8 eV**. We find the electron cylinder has an exponent of $n = 4.7$ and the hole cylinder $n = 4.96$. Hence for our model we choose $n = 5$.

interatomic distances, similar to behaviour seen in $YFe_2Ge_2$ (ref.[9]). Note since $u$ is defined in the range of $[-\pi, \pi]$, the final areas must be rescaled according to the real extent of the $\vec{e}_z$ direction.

With these parametric equation defined, the simulated Fermi surface can be obtained by defining the surface

$$\vec{S} = \vec{q} + \vec{p} + \vec{A} \tag{6}$$

where

$$\vec{q} = (q_x, q_y, q_z), \tag{7}$$

and

$$\vec{p} = (p_x, p_y, p_z). \tag{8}$$

$\vec{A}$ is an offset from the centre of the Brillouin zone such that the cylinders are centred at the **X** and **Y** high-symmetry points. For these simulations the unit-cell was assumed to have dimen-

sions:[10]

$$
\begin{aligned}
a &= 4.123 \text{ Å}, \\
b &= 6.086 \text{ Å}, \\
c &= 13.812 \text{ Å}.
\end{aligned} \tag{9}
$$

The 0° dHvA data fix the areas of each cylinder at constant values $\mathcal{A}_{h/e}$ while the symmetry properties of the Brillouin zone allows the hole-like cylinder to only be warped in the $\vec{e}_x$ direction, whereas for the electron-like cylinder warping is only allowed in the $\vec{e}_y$ direction. This means that once the area for each cylinder has been determined there are only two free parameters that can be varied to fit the data:

$$
\frac{R_a}{R_b}, \quad \text{where} \quad R_{a_{e/h}} R_{b_{e/h}} = \mathcal{A}_{e/h}, \tag{10}
$$

$$
w_{a/b} \quad \text{for} \quad e/h. \tag{11}
$$

For the hole-like cylinder, values of:

$$
\begin{aligned}
R_a &= 1.92, \\
R_b &= 2.12, \\
w_a &= 0.52, \\
w_b &= 0
\end{aligned} \tag{12}
$$

were determined, whereas for the electron-like cylinder it was found that:

$$
\begin{aligned}
R_a &= 1.55, \\
R_b &= 2.59, \\
w_a &= 0, \\
w_b &= -0.15.
\end{aligned} \tag{13}
$$

With these parameters, Fermi surfaces were generated and visualised using PyVista.[5] Simulated frequencies were determined according to a similar methodology to SKEAF,[7] although the closed-cylindrical topology of each surface makes determining extremal frequencies significantly easier. Since there is only one warping parameter, each cylinder can contribute at most only two extremal areas. Extremal areas were determined by shifting each cylinder to the origin and creating a supercell of the cylinder (extending 20 Brillouin zones). Slicing planes were then placed at regular intervals along the $\vec{c}$ direction where the angle of the slicing planes could be varied continuously through 90° towards either the $\vec{a}$ or $\vec{b}$ direction. The maximal and minimal areas, $\mathcal{A}$, of the intersection of each slicing plane with each cylinder could then be determined, and hence the frequency contribution, $f$, of each cylinder was calculated according to the Onsager relation,[11]

$$f = \frac{\hbar \mathcal{A}}{2\pi e}. \tag{14}$$

It should be noted that the quantum oscillation data is degenerate with respect to inversions of the warping parameter. This is to say that making $w_{a/b}$ negative will invert the warping of the cylinders while leaving the simulated oscillation pattern the same (see Supplementary Figure S11). In this work we chose the warping to best emulate the Fermi surfaces seen for U = 8 eV as used in several other $UTe_2$ works, in addition to studies of several uranium oxides.[12–14]

## Inverted-warping Fermi surface

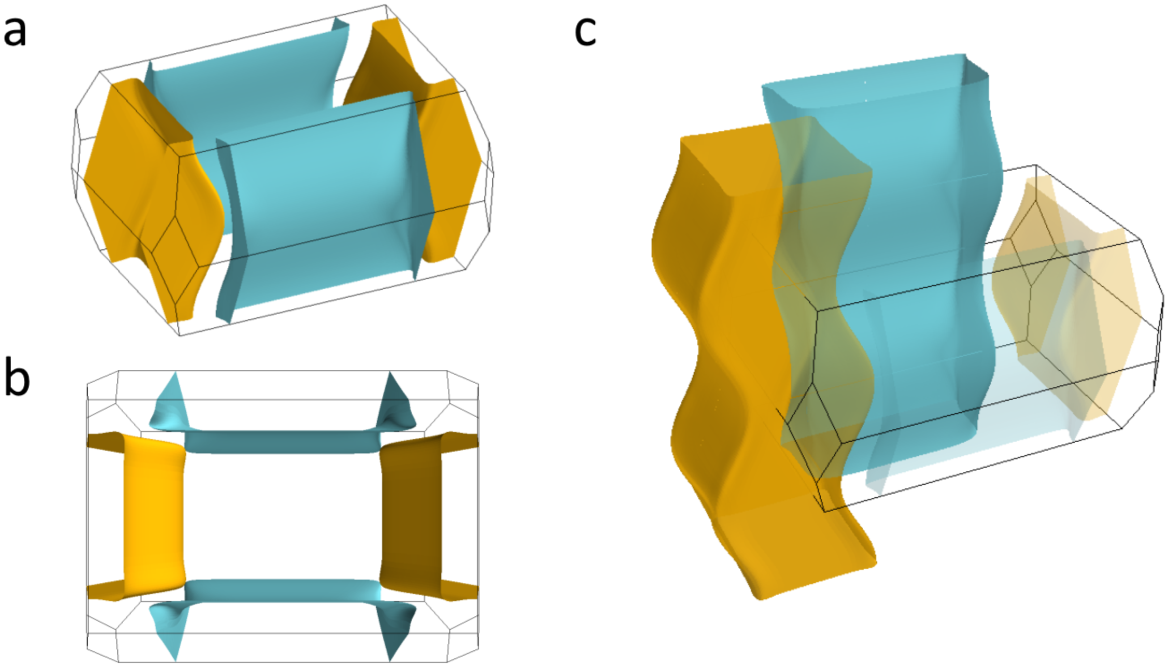


**Fig. S11. An example degenerate Fermi surface. a,** Side-on view of the Fermi surface. **b,** Top-down view of the Fermi surface. **c,** Extension of cylinders outside of the first Brillouin zone. When the warping parameters are inverted an alternate Fermi surface is produced with simulated dHvA effect frequencies that identically match the quantum oscillation data (see Fig. 4 of main text). The shape of these surfaces more closely resembles the DFT-generated Fermi surfaces of U = 1.7-2.0 eV than those of other U parameters.

## Quantum oscillations in the contactless resistivity of $UTe_2$

In addition to our dHvA effect study presented in the main text, we also measured quantum oscillations from the Shubnikov-de Haas (SdH) effect. Contactless resistivity was measured by utilizing a proximity diode oscillator (PDO) circuit, with the change in frequency of this circuit as a function of magnetic field being related to the change in resistivity of a $UTe_2$ crystal (for further details of this measurement technique, see for example refs.[15–17]).

Figure S12 shows the contactless resistivity of $UTe_2$ for field oriented along the $\vec{c}$ direction (purple curves) and at a tilt angle of 51° away from $\vec{c}$ towards $\vec{b}$ (green curves). For field aligned along $\vec{c}$, a monofrequency oscillatory signal is clearly resolved, with no other frequency peaks resolvable above the noise floor. This is in very good agreement with the torque data presented in the main text.

We note that a recent field-modulation technique study of the dHvA signal of $UTe_2$ along the $\vec{c}$ direction reported multiple frequency branches for this orientation.[18] The authors posited that the disagreement between their result and that of our torque study is likely due to a misalignment in one of the experiments. We note that our employed cantilever beam magnetometry technique of capacitive torque magnetometry in general allows for better accuracy and precision in angular orientation than magnetic torque performed using piezoelectric cantilevers, due to the larger sample and cantilever sizes involved that enable easier orientation during the mounting procedure. However, by the nature of the measurement, the possibility of some non-negligible deflection of the cantilever away from the equilibrium position of alignment – although unlikely due to the very small magnitude of background torque close to a high symmetry direction – nonetheless cannot be entirely excluded. For our contactless resistivity study presented here, we replicate the monofrequency waveform of Fig. 3 of the main text very well. We note that the alignment of the crystal for the PDO experiment was assisted by choosing a platelet shaped sample, with a dominant (001) face. This was then secured onto a planar PDO coil such that

the base plate of the measurement coil – the plane of which runs orthogonally to the crystallographic $\vec{c}$ direction – could be firmly secured onto the rotator platform of the measurement probe. With this platform oriented normal to the applied field, the field is then aligned along the $\vec{c}$ direction. Angular orientation was calibrated by use of a Hall sensor.

We note that the resolution of our PDO measurement was not as sensitive as the comparative torque measurement at $\phi = 0^\circ$ in the main text, as the second harmonic is not discernible. This may be due to some technical differences in the optimisation of the two measurement techniques, or due to a difference in sample quality, or a combination of both. Despite the lower temperature of the $51^\circ$ measurement, it is not sensitive enough to resolve any frequency components. This is consistent with the rapidly increasing frequency profile of our quasi-2D Fermi surface model, accompanied by a sharp diminution of oscillatory amplitude, as the field is titled further away from the axis of the cylinders (as demonstrated by Fig. 2 of the main text) – thus, the oscillatory amplitude appears to have fallen below the comparatively high noise floor of the PDO measurement. Our SdH measurements therefore do not indicate the presence of any other Fermi sheets beyond the two quasi-2D cylindrical sections that are captured by our Fermi surface model and presented in Fig. 4 of the main text.

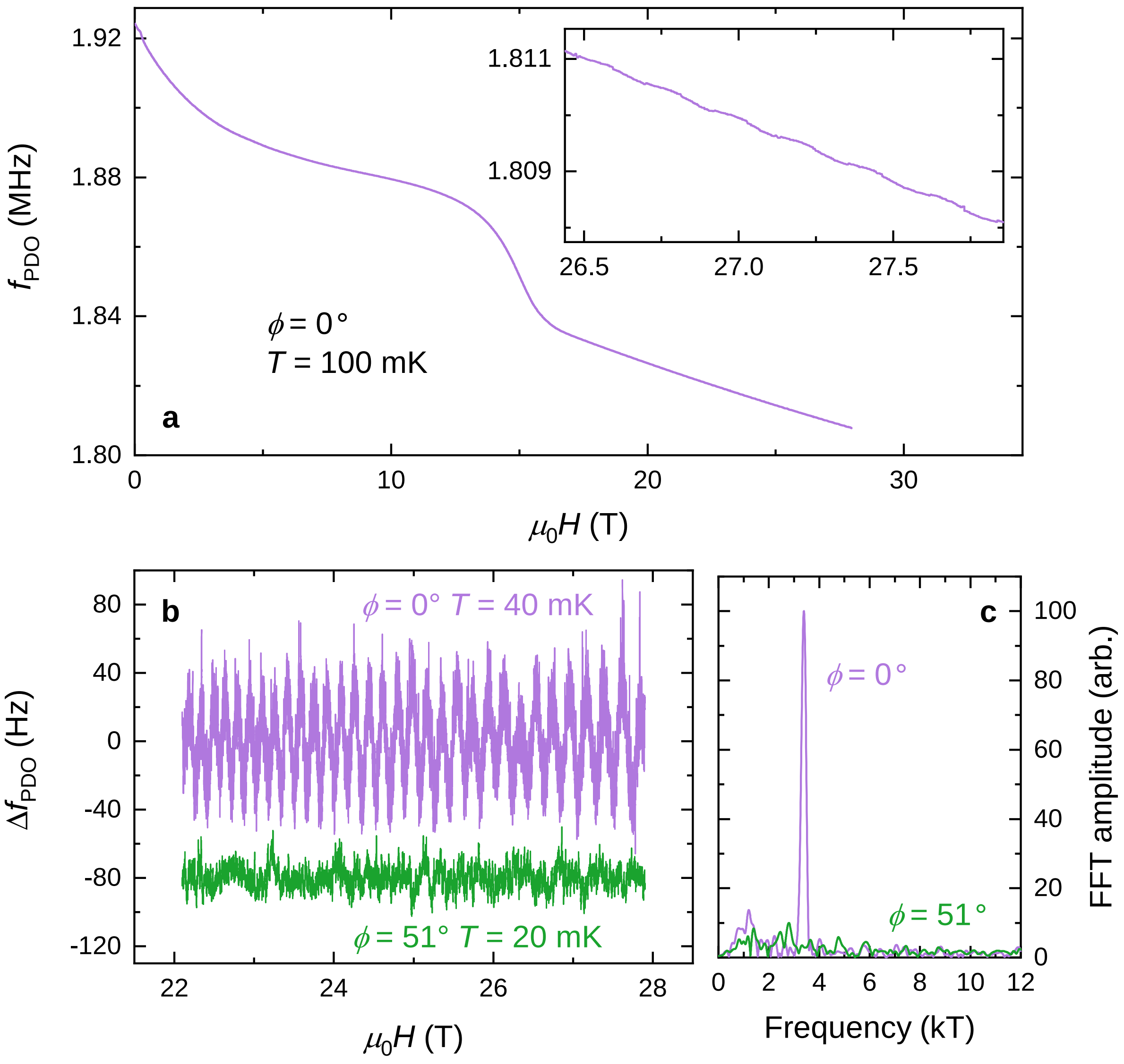


**Fig. S12. Quantum oscillations in the contactless resistivity of UTe$_2$. a**, PDO frequency for a coil connected to a UTe$_2$ crystal with magnetic field oriented along the $\vec{c}$ direction. (Here $\phi$ is defined as the angle between $\vec{c}$ and $\vec{b}$.) The inset gives a zoomed view at high field, showing that the 3.5 kT oscillations are clearly visible in the raw signal before background subtraction. **b**, Background-subtracted PDO signal for magnetic field oriented along the $\vec{c}$ direction ($\phi = 0°$) and tilted 51° away from $\vec{c}$ towards $\vec{b}$. The 51° data has been offset by 80 Hz for clarity. The subtraction procedure for both angles was performed identically. **c**, FFT spectra of the data in panel (b).

## Estimation of the mean-free path

We can estimate a lower bound for the mean-free path of the samples investigated in this study by considering the (real-space) cyclotron orbits that give rise to the observed magneto-quantum oscillations. Assuming an approximately circular cross-sectional surface area normal to the applied magnetic field, then $\mathcal{A} = \pi k_F^2$, where $k_F$ is the Fermi wave-vector. Consider now the cyclotron motion of an electrically charged quasiparticle in the presence of a magnetic field $B$, which may be described by

$$m^* \frac{v_F^2}{r} = eBv_F, \tag{15}$$

where $r$ is the radius of the cyclotron orbit and $v_F$ is the Fermi velocity. As $\hbar k_F \equiv m^* v_F$, we can substitute this into the above and with use of Eqn. 14 we may express $r$ as

$$r = \sqrt{\frac{2\hbar f}{eB^2}}. \tag{16}$$

Therefore, the observation of a quantum oscillation frequency of 18.5 kT (in Fig. 2 of the main text) at a magnetic field of 26 T implies a cyclotron orbit of radius ≈ 1900 Å. Thus, this gives an approximate lower bound on the mean-free path of the sample.

We can compare this value deduced from cyclotron orbit arguments with an estimation of the mean-free path, $\lambda$, expected from Drude theory.[19] For a metal with carrier density $n$, $\lambda$ may be expressed as:

$$\lambda = \frac{m^* v_F}{ne^2 \rho_0} = \frac{\hbar k_F}{ne^2 \rho_0}. \tag{17}$$

Taking $n$ from a prior Hall effect study,[20] which found for $B < 35$ T that $n = 1.6 \times 10^{22}$ cm$^{-3}$, then a sample with $\rho_0 \lesssim 0.5$ μΩ cm (Fig. 1b) has $\lambda \sim 2000$ Å. Thus, this approximation underlines the pristine quality of the $UTe_2$ single crystals investigated in this study.

## Conductivity tensor analysis

The absence of a pronounced anisotropy of the electrical conductivity of $UTe_2$ has been proposed as evidence in favour of the existence of a 3D Fermi surface pocket in this material.[21] This is because in many materials with Fermi surfaces consisting exclusively of quasi-2D cylindrical sections, such as cuprates and ruthenates, the resistivity in the plane orthogonal to the cylindrical axis is typically orders of magnitude lower than for transport parallel to the axis.[22–24] However, the Fermi surface cylinders of typical cuprates and ruthenates do not have the pronounced warping that we find is required to describe the dHvA data of $UTe_2$ (Fig. 4 of the main text). Therefore, the vector normal to their Fermi surfaces – at all points on the surfaces – never has a significant component in the $k_z$ direction. By comparison, we show below that our Fermi surface model of $UTe_2$ – with its strongly pronounced undulations – naturally accounts for the isotropy of the electrical conductivity tensor, as we plot in Fig. 5 of the main text.

The conductivity of a metal within the relaxation time approximation[25] can be written as:

$$\sigma^{\alpha\beta}(T) = \frac{1}{k_B T} \int_{-\infty}^{\infty} f(1-f)\mathbf{X}^{\alpha\beta}(\epsilon) d\epsilon \tag{18}$$

for energy $\epsilon$, temperature $T$, where $k_B$ is Boltzmann's constant and $f$ is the Fermi-Dirac distribution function

$$f = \left(\exp\left[\frac{(\epsilon-\mu)}{k_B T}\right] + 1\right)^{-1}, \tag{19}$$

for chemical potential $\mu$, and $\mathbf{X}^{\alpha\beta}(\epsilon)$ is the transport coefficient[25] defined to be

$$\mathbf{X}^{\alpha\beta}(\epsilon) = 2e^2 \int_{\mathbf{k}} \sum_i v_i^\alpha(\mathbf{k}) v_i^\beta(\mathbf{k}) \tau_{i,\mathbf{k}} \delta(\epsilon - \epsilon_i(\mathbf{k})) \frac{d\mathbf{k}}{8\pi^3}. \tag{20}$$

Here $v_i^\alpha(\mathbf{k})$ is the group velocity of a quasiparticle:

$$v_i^\alpha(\mathbf{k}) = \frac{1}{\hbar}\frac{\partial \epsilon_i}{\partial \mathbf{k}} \tag{21}$$

where $i$ is the band index, $\alpha \in [x, y, z]$ and $\tau_{i,\mathbf{k}}$ is the quasiparticle relaxation time.

In the limit that $T \rightarrow 0$

$$\frac{f(1-f)}{k_{\mathrm{B}}T} \rightarrow \delta(\epsilon - \mu). \tag{22}$$

Therefore, we can redefine the conductivity as:

$$\sigma^{\alpha\beta}(T) \propto \mathbf{X}^{\alpha\beta}(\mu) \tag{23}$$

$$\propto \int_{\mathbf{k}} \sum_i v_i^{\alpha}(\mathbf{k}) v_i^{\beta}(\mathbf{k}) \tau_{i,\mathbf{k}} \delta(\epsilon - \mu) \frac{d\mathbf{k}}{8\pi^3} \tag{24}$$

$$\propto \int_{\mathbf{k}_f} \sum_i v_i^{\alpha}(\mathbf{k}_f) v_i^{\beta}(\mathbf{k}_f) \tau_{i,\mathbf{k}_f} \frac{d\mathbf{k}_f}{8\pi^3} \tag{25}$$

where $\mathbf{k}_f$ is now the set of vectors which lie on the Fermi surface. In the constant relaxation time approximation this can be further simplified to

$$\sigma \propto \sum_{\mathbf{k}_f} \sum_i v_i^{\alpha}(\mathbf{k}_f) v_i^{\beta}(\mathbf{k}_f) \tag{26}$$

where $\sum_{\mathbf{k}_f}$ is a sum over all $\mathbf{k}_f$ on the Fermi surface.

Since the empirical Fermi surface in this model does not contain information about the energy at each $k$-point, we approximate $v_i^{\alpha}(\mathbf{k}_f) \sim n_i^{\alpha}(\mathbf{k}_f)$, the normal to the Fermi surface. Therefore, as the system is divided into cells of non-equal area the conductivity becomes

$$\sigma \propto \sum_j \sum_i A_{i,j}^2 n_{i,j}^{\alpha} n_{i,j}^{\beta} \tag{27}$$

where $\sum_j$ is a summation over the cells of the geometrical surface that defines the Fermi surface and $A_{i,j}$ is the area of the $j^{\text{th}}$ cell of the $i^{\text{th}}$ band.

Applying this formalism directly to our simulated Fermi surface of $UTe_2$ gives the results presented in Figure S13(a,b). When no warping is included, the $\sigma^{zz}$ component is several orders of magnitude less than $\sigma^{xx}$ and $\sigma^{yy}$. Increasing the warping towards the level used to fit the quantum oscillation data (warping = 100%) rapidly increases $\sigma^{zz}$ by several orders of magnitude. Further increasing the warping beyond 100% only serves to slightly increase the proportional contribution of $\sigma^{zz}$ to the overall conductivity matrix.

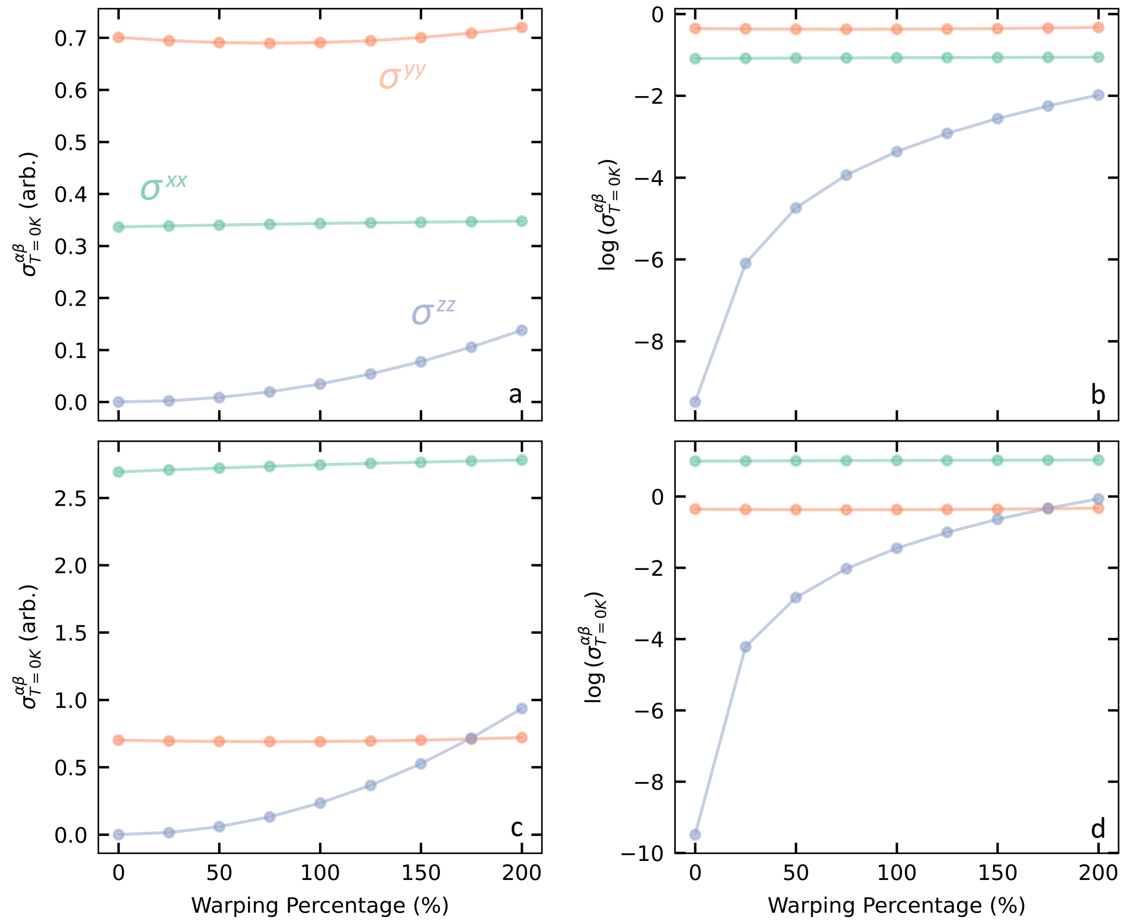


**Fig. S13. Diagonal components of the conductivity tensor as a function of Fermi surface warping. a,** Components of the conductivity tensor determined directly from the geometrical Fermi surface fitting. 100% warping corresponds to the extent of undulation required to fit the dHvA data (Fig. 4 of the main text). **b,** The same components plotted on a logarithmic scale showing the rapid increase in $\sigma^{zz}$ as the cylindrical warping is increased. **c,** Components of the conductivity tensor including the (directional-dependent) mean free path (details specified in the text). **d,** The same components plotted on a logarithmic scale showing that at 100% warping all components of the conductivity are of the same order of magnitude.

Therefore, this simple geometrical analysis of the dependence of the electrical conductivity tensor on the extent of undulations along the Fermi surface cylinders very well captures the empirical observation of the absence of pronounced anisotropy in the conductivity of $UTe_2$. However, we note that this simple treatment predicts $\sigma^{yy} > \sigma^{xx}$ for all warping parameters, i.e. independent of the cylindrical undulations. This is at odds with the experimental data reported

in Eo et al.[21] in which $\sigma^{xx} \approx 2\sigma^{yy}$ and $\sigma^{yy} \approx \sigma^{zz}$. We note that in our approach outlined above we made the simplifications of taking $\tau_{i,\mathbf{k}}$ to be isotropic and constant and $v_i^{\alpha}(\mathbf{k}_f)$ to be $\sim n_i^{\alpha}(\mathbf{k}_f)$ whereas in reality it varies as $\frac{\partial \epsilon_i}{\partial \mathbf{k}}$. This simplified our treatment in order to focus exclusively on the dependence of the conductivity to the shape of the Fermi surface – the primary aim of this analysis.

However, to effectively capture the additional effect of possible anisotropies in the directional dependencies of the quasiparticle velocity or the relaxation time – which would in turn result in a (directional-dependent) renormalized mean free path that may be important for considering the conductivity in the $xy$-plane – we must expand our simple geometrical model to explicitly account for this possible $\mathbf{k}$-dependence of the mean free path. From Ziman (ref.[26]) we may conveniently re-express the electrical conductivity as

$$\sigma^{\alpha\beta}(\omega) = \frac{e^2}{4\pi^3\hbar}\frac{\tau(\epsilon_{\mathrm{F}})}{1 - i\omega\tau(\epsilon_{\mathrm{F}})}\int \frac{v^{\alpha}(\mathbf{k})v^{\beta}(\mathbf{k})}{|\mathbf{v}(\mathbf{k})|}d\mathcal{S} \tag{28}$$

for frequency $\omega$ where $d\mathcal{S}$ denotes integration over the Fermi surface. Then generalizing to a $\mathbf{k}$-dependent relaxation rate for $\omega = 0$ and $\alpha = \beta$ Eqn. 28 becomes

$$\sigma^{\alpha\alpha} = \frac{e^2}{4\pi^3\hbar}\int \frac{\tau(\mathbf{k})v^{\alpha}(\mathbf{k})^2}{|\mathbf{v}(\mathbf{k})|}d\mathcal{S} \tag{29}$$

$$= \frac{e^2}{4\pi^3\hbar}\int \lambda^{\alpha}(\mathbf{k})n^{\alpha}(\mathbf{k})d\mathcal{S} \tag{30}$$

where $\lambda^{\alpha}(\mathbf{k}) = v^{\alpha}(\mathbf{k})\tau(\mathbf{k})$ is the mean free path and $n^{\alpha}(\mathbf{k}) = \frac{v^{\alpha}(\mathbf{k})}{|\mathbf{v}(\mathbf{k})|}$. This form is convenient as mass renormalization due to soft modes cancels in the product of the velocity and scattering time (i.e. cancels both in $\lambda^{\alpha}(\mathbf{k})$ and in $n^{\alpha}(\mathbf{k})$).

Incorporating the additional degree of freedom of $\lambda^{\alpha}(\mathbf{k})$ from Eqn. 30 as a free fitting parameter into our geometrical $UTe_2$ conductivity model, we can now excellently capture the results of Eo et al.[21] as we plot in Fig. S13(c,d) and Fig. 5 of the main text. While the inclusion

of $\lambda^{\alpha}(\mathbf{k})$ has only a slight effect on the proportional contribution of $\sigma^{zz}$ to the overall conductivity matrix – an important aspect of this analysis in the context of confidently determining the Fermi surface geometry of $UTe_2$ – it does indeed reconcile the factor of $\approx 2$ variation between $\sigma^{xx}$ and $\sigma^{yy}$ reported in ref.[21]

We remark that, independent to our primary discussion concerning the relationship between the conductivity tensor and Fermi surface undulation of $UTe_2$, our finding here of a pronounced variation of the mean free path in the $k_x$-$k_y$ plane – which is required in order to reconcile simple bandstructure considerations with empirical observations – is itself an interesting point worthy of further exploration. If one were to somewhat naïvely attempt to predict the anisotropy of $\lambda$ in the $k_x$-$k_y$ plane by only considering bandstructure calculations from e.g. ref.[27] then since the velocity for the Te-$p$ bands that dominate the contribution to $\sigma_{yy}$ is expected to be lower than for the U-$d$ bands that contribute to $\sigma_{xx}$, one would expect that $\sigma^{yy} > \sigma^{xx}$ – the opposite of what is observed experimentally by Eo et al.[21]

We propose that a possible mechanism for the mean-free path anisotropy in the $k_x$-$k_y$ plane of $UTe_2$ arises due to enhanced scattering between Te-$p$ sheets in the $k_y$ direction relative to the U-$d$ sheets in the $k_x$ direction (Figure S14). As pointed out by Xu et al.,[12] the density of states of the Te-$p$ electrons at the Fermi level is expected to be higher than that of the U-$d$ states. Therefore the spin or charge susceptibility $\chi(\mathbf{q})$ at $\mathbf{q} = \mathbf{Q_a}$ will be higher than that at $\mathbf{q} = \mathbf{Q_b}$. Subsequently, there will be greater large-angle scattering for charge carriers in the $y$ direction than the $x$ direction, which will suppress the mean-free path and hence the conductivity in the $y$ direction, thereby naturally accounting for the observation that $\sigma_{xx} > \sigma_{yy}$. We note that such a mechanism may have important implications regarding the likely pairing state of the spin-triplet superconductivity, and is worthy of further study beyond the scope of this analysis.

As a final note, we stress that the key result of this analysis is that the presence of pronounced axial undulations along the cylindrical Fermi surface sheets is sufficient to account for

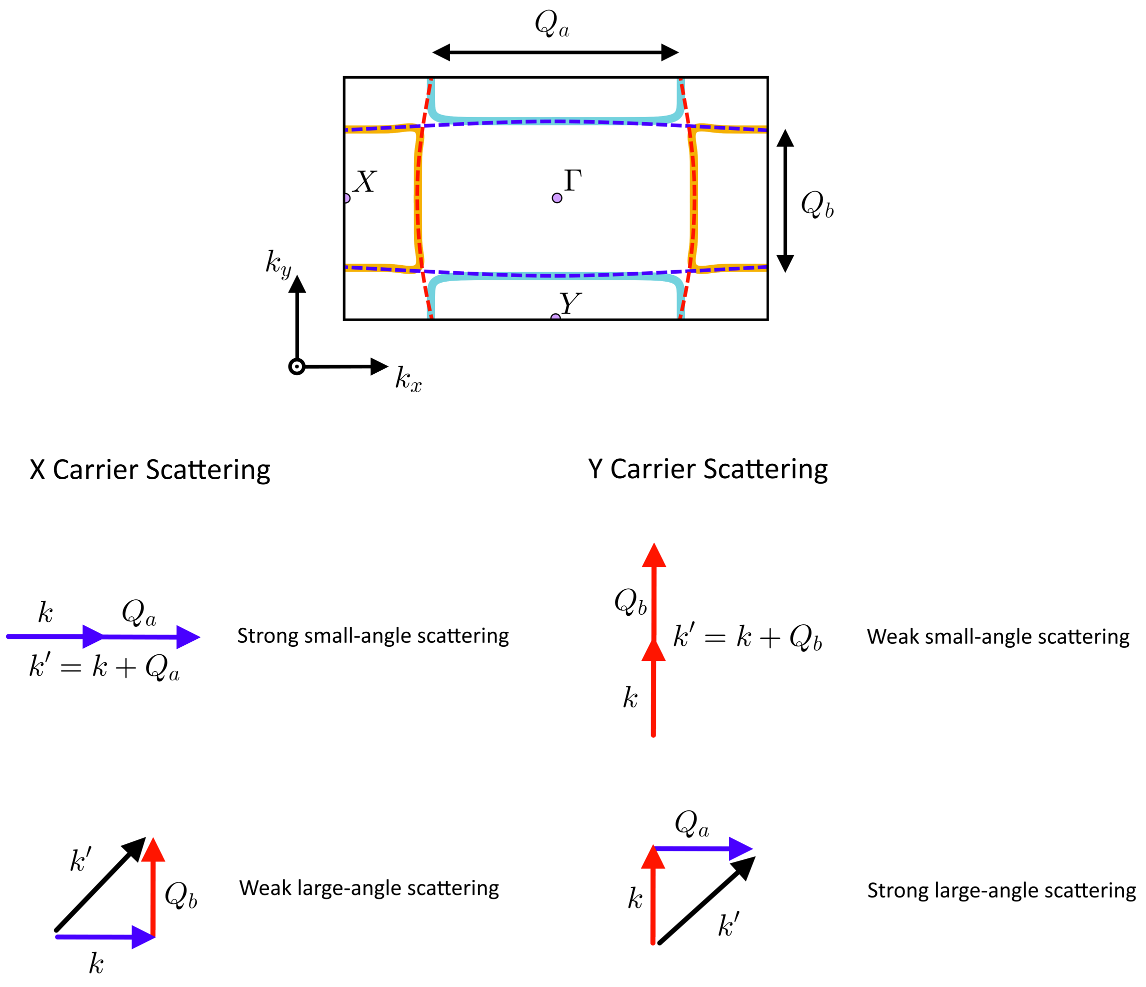


**Fig. S14. Schematic of charge carrier scattering in the $x$ and $y$ directions.** Enhanced charge and spin susceptibility at $\mathbf{Q_a}$ and $\mathbf{Q_b}$ results in greater large-angle scattering of charge carriers moving in the $y$ direction than in the $x$ direction, whereas the effect of small-angle scattering is ineffective in reducing the current. Subsequently the mean free path of carriers in the $y$ direction is reduced relative to the $x$ direction. This acts to suppress $\sigma_{yy}$ resulting in $\sigma_{xx} > \sigma_{yy}$.

the lack of anisotropy in the electrical conductivity tensor of $UTe_2$. This results simply from considering the extent of undulations along the Fermi surface cylinders, and is independent of higher order considerations such as the possible anisotropy of the mean free path in the $k_x$-$k_y$ plane. Our analysis of the electrical conductivity tensor demonstrates that the presence of any

3D Fermi surface section(s) are not required in order to account for the reported conductivity data; however, as for fitting to the dHvA data, the presence of pronounced undulations along the cylindrical axes is essential.

## Identification of two distinct Fermi surface sections contributing to the observed oscillatory waveform

The high resolution of the quantum oscillation data measured in this study allows us to perform a detailed quantitative analysis, to better understand the geometrical properties of the $UTe_2$ Fermi surface. The arguments presented in the main text, and the discussion of our Fermi surface simulation given above, present a strong argument in favour of the $UTe_2$ Fermi surface being composed of two quasi-2D cylindrical sheets. Prior analyses of cylindrical Fermi surfaces in hole-doped cuprates have carefully considered the amount of corrugation that may be present along the sides of the cylinders,[28–31] which can have important implications for Fermi surface nesting vectors.[32,33]

Our simulated Fermi surface (Fig. 4 of the main text) possesses pronounced undulations along the length of the cylinders. However, on a smaller scale, our model predicts that the cylinders are very smooth, with negligible crinkles or corrugations along the surfaces. Furthermore, the model expects a singular frequency component to be observed for field along $\vec{c}$. This would imply that both cylinders have the same cross-sectional area, and again, that there are only negligibly small ripples or corrugations up their lengths – only the large-scale undulations, that go through one complete period of undulation for each Brillouin zone, without adding additional extremal orbits for field along $\vec{c}$.

To assess the validity of these geometrical assumptions, we can closely inspect the dHvA signal for field applied collinear to the cylindrical axes (the $\vec{c}$ direction). We note that our measurement has an angular uncertainty of approximately 2° in this rotation plane. We note further that the amplitude of the background magnetic torque was minimal at 0°, as expected (although, the amplitude of the oscillatory component was maximal). Due to the dilution refrigerator still cooling back to base temperature after eddy-current-induced heating was introduced by sweeping up quickly (0.5 T/min) from 0 to 28 T, which slightly diminished the amplitude

of the highest-field section of the data for $\theta = 0^\circ$ in Fig. 2 of the main text upon sweeping down slowly, here we consider just the data obtained for $\mu_0 H \leq 26.5$ T (as we also do for the LK analysis in the main text), to mitigate this spurious effect. For the general case of two cylindrical Fermi sheets, labelled $\alpha$ and $\beta$, of unspecified cross-sectional area and degree of corrugation, the theoretically expected[28,29,34,35] quantum oscillatory waveform for magnetic field, $H$, applied parallel to their axes may be approximated as:

$$\Delta\tau = \sum_{n=\alpha,\beta} \Delta\tau_{n,0} \cdot R_{\mathrm{T}} R_{\mathrm{D}} \cdot J_0\left(\frac{2\pi\Delta f_n}{\mu_0 H}\right) \cdot \cos\left(\frac{2\pi f_n}{\mu_0 H}\right), \tag{31}$$

where $\Delta\tau_0$ is the amplitude in the infinite-field limit, $R_{\mathrm{T}}$ is the temperature damping coefficient (computed from the data presented in Fig. 3 of the main text), $R_{\mathrm{D}}$ is the Dingle damping coefficient[36] of form $R_{\mathrm{D}} = \exp\left(\frac{-D_n}{\mu_0 H}\right)$ for damping factor $D_n$, $\Delta f_n$ is the depth of corrugation (in frequency-space) of the $n^{\mathrm{th}}$ Fermi sheet, and $f_n$ corresponds to the oscillatory frequency corresponding to a cross-sectional area $\mathcal{A}_n$ by the Onsager relation.[11] $J_0$ denotes a zeroth order Bessel function of the first kind, to capture the extent of any possible corrugation along the lengths of the cylinders, which would result in interference due to phase smearing.

We perform an unconstrained fit to Eqn. 31 in Fig. S9, which yields values of $f_\alpha = 3470(15)$ T, $\Delta f_\alpha = 13(4)$ T, $f_\beta = 3485(13)$ T, and $\Delta f_\beta = 7(6)$ T. This implies that both cylinders have identical cross-sectional areas (within uncertainty), and that the presence of corrugation is negligible as ${}^{\Delta f_\alpha}/{}_{f_\alpha} = 0.004$. Therefore, we conclude that the description provided by our Fermi surface simulation – of quasi-2D cylindrical sections with identical cross-sectional areas and negligible small-scale corrugations – is well supported by this analysis.

We note that this treatment is only approximate as it is assumes cylinders having circular cross-sections, rather than the super-elliptical cross-sections we find in our Fermi surface simulations – hence, we have restricted this analysis solely to the 0° data. A similar analysis at inclined angles, beyond the scope of this work, fully accounting for the squircular nature of the

cylinders, could be illuminating in evaluating the effects of a possible Yamaji angle in the vicinity of the orientation at which the very high magnetic field re-entrant superconducting phase is located[37,38] (see Fig. S22).

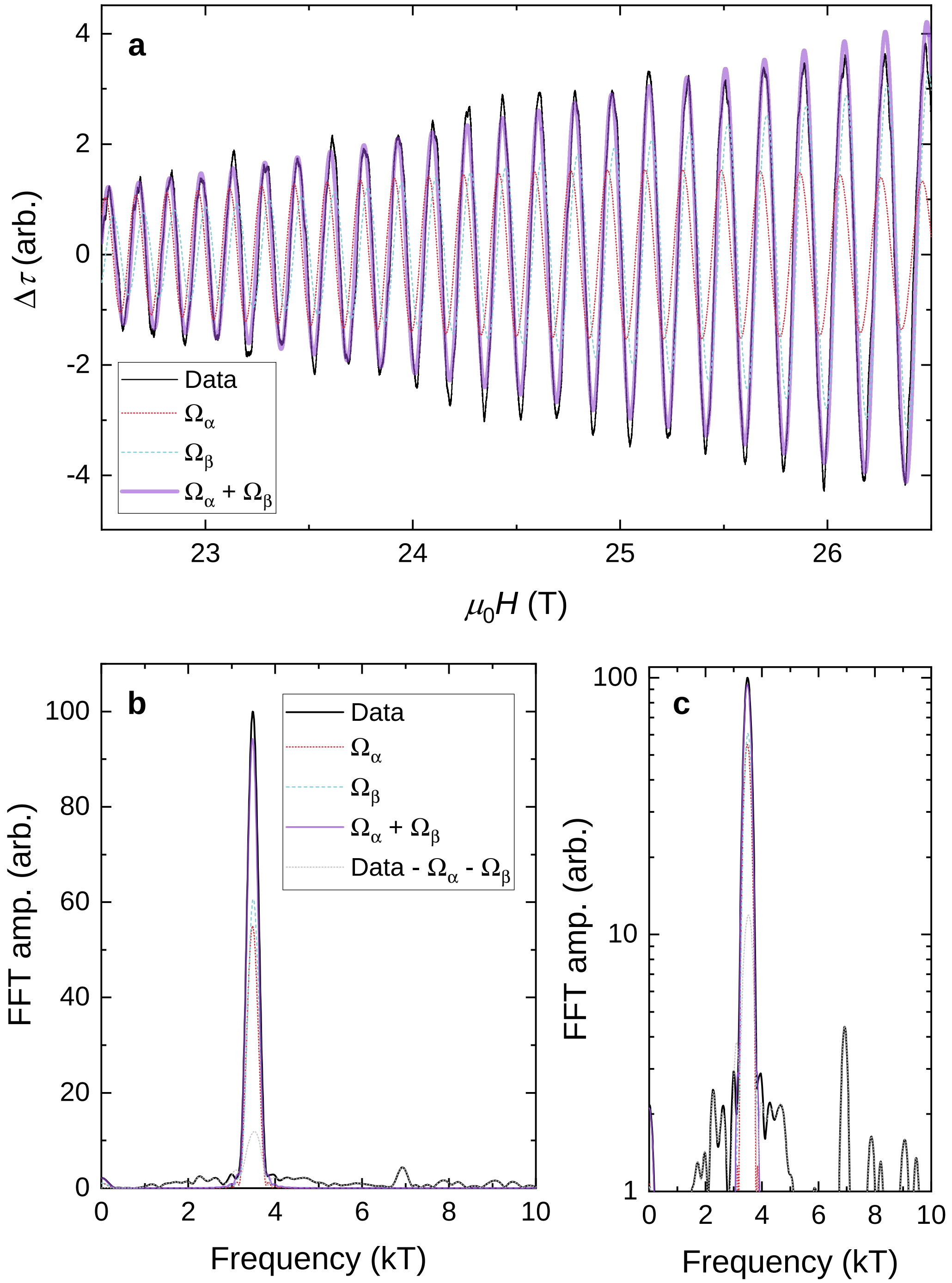


**Fig. S15. Isolating the dHvA contributions of two quasi-2D Fermi surface sections of equal area. a**, The 19 mK dHvA data from Fig. 3 of the main text, for magnetic field oriented along $\vec{c}$, is reproduced here (black curve). Red solid and blue dashed curves are the $\alpha$ and $\beta$ components, respectively, of Eqn. 31, obtained by performing an unconstrained fit to the data. $\Omega_{\alpha,\beta}$ represent the oscillatory contribution from each of the two distinct cylindrical Fermi surface sections. The purple curve is the sum of these two components ($\Omega_\alpha + \Omega_\beta$), which fits the measured data very well. **b**, FFTs of the curves in (a), along with the FFT of the residual curve obtained by subtracting the fit from the data (grey dashed line).

**Fig. S15. (cont.) c**, The same FFT spectra as (b) plotted here on a logarithmic amplitude axis. No clear frequency peaks are distinguishable from the noise after subtracting the dominant component, except for the second harmonic at 7.0 kT. Therefore, the two cylindrical sections (labelled here as $\alpha$ and $\beta$) appear to be the sole Fermi surface sections with closed cyclotron orbits normal to $\vec{c}$.

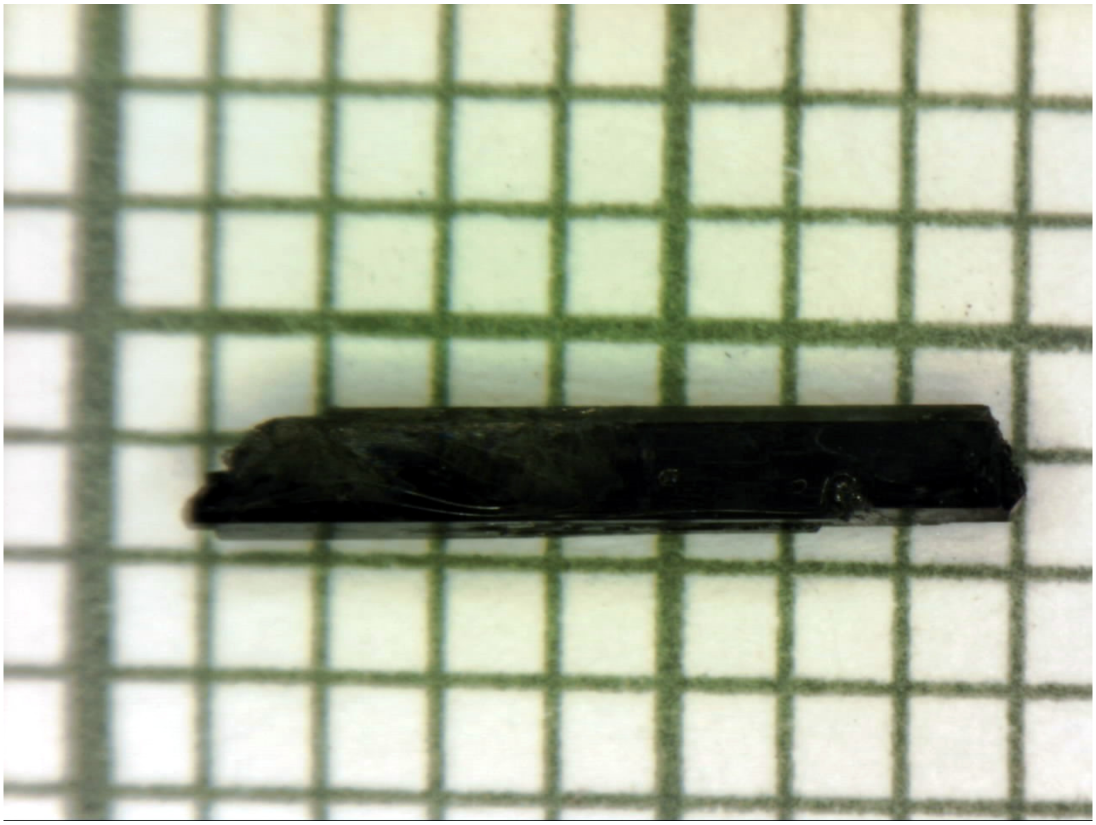

**Fig. S16. $UTe_2$ single crystal.** Photograph of a typical $UTe_2$ crystal prepared by the molten salt flux method. Our $UTe_2$ samples tend to crystallise to have flat (001) and (011) surfaces, with the direction of longest extent (left-right in this image) being the $\vec{a}$ direction.

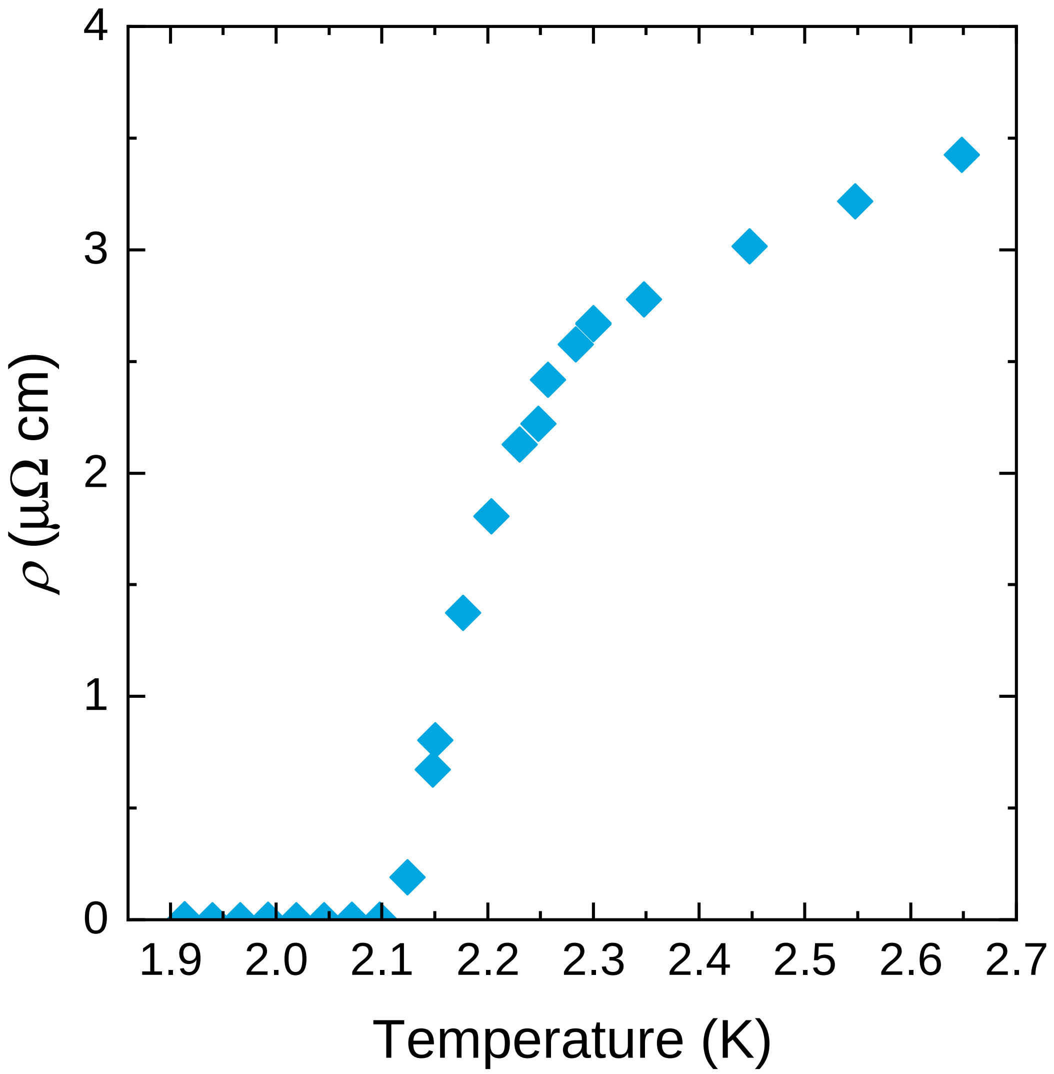


**Fig. S17. Determining $T_c$ from resistivity measurements.** The same electrical resistivity data as that in Fig. 1 of the main text, here plotted linearly in temperature close to the superconducting transition. A $T_c$ of 2.1 K is clearly resolved, as determined by zero resistivity (defined as being below the detection limit of 0.01 μΩ cm).

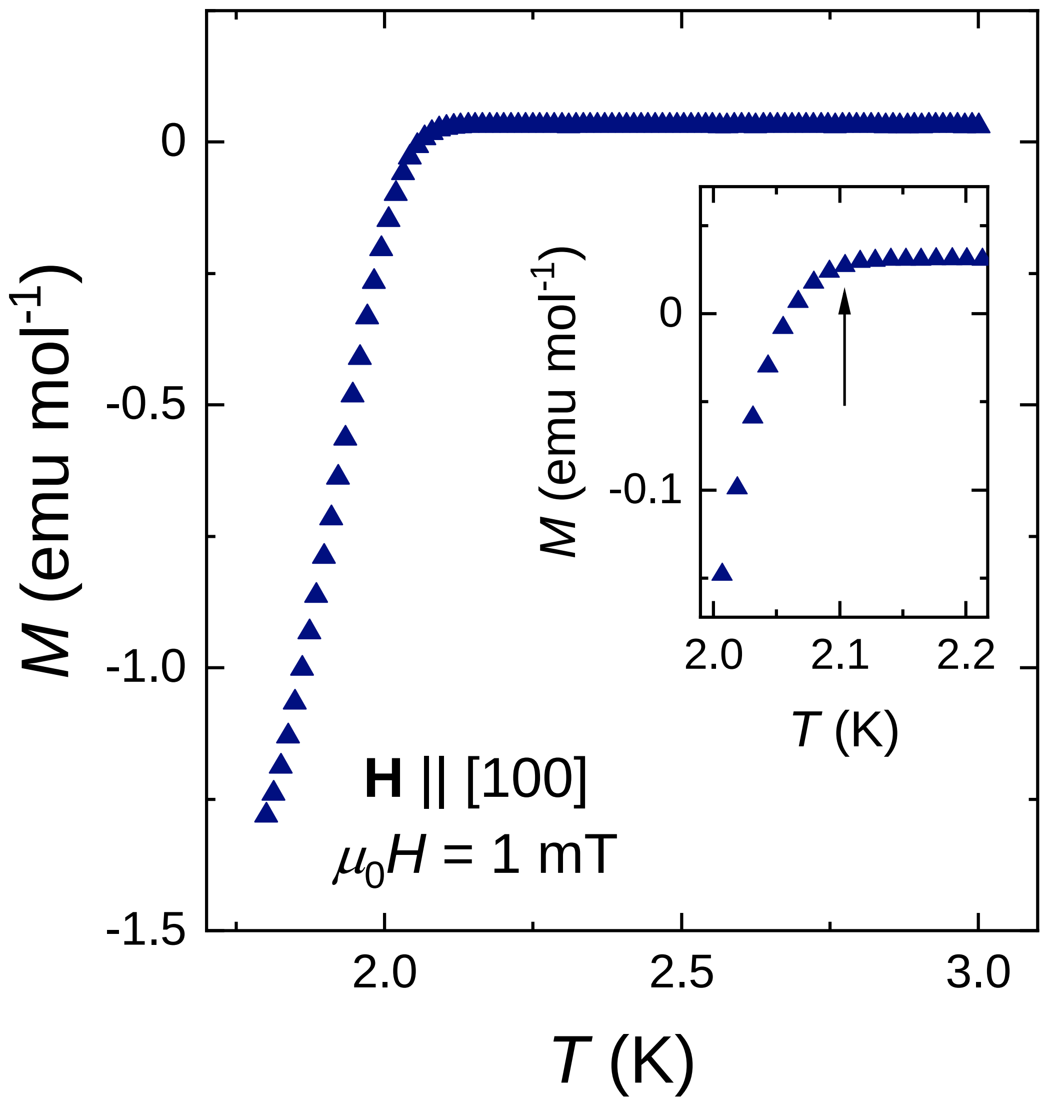


**Fig. S18. Determining $T_c$ from magnetisation measurements.** dc magnetisation, $M$, of a $UTe_2$ sample from the same growth batch as those used in our quantum oscillation measurements. The onset of superconductivity is clearly resolved at $T$ = 2.1 K, indicating a single bulk transition at this temperature. A small magnetic field of 1 mT was applied along the $\vec{a}$ direction; the sample was affixed to a quartz sample holder by cryogenic varnish and measured in a Quantum Design MPMS.

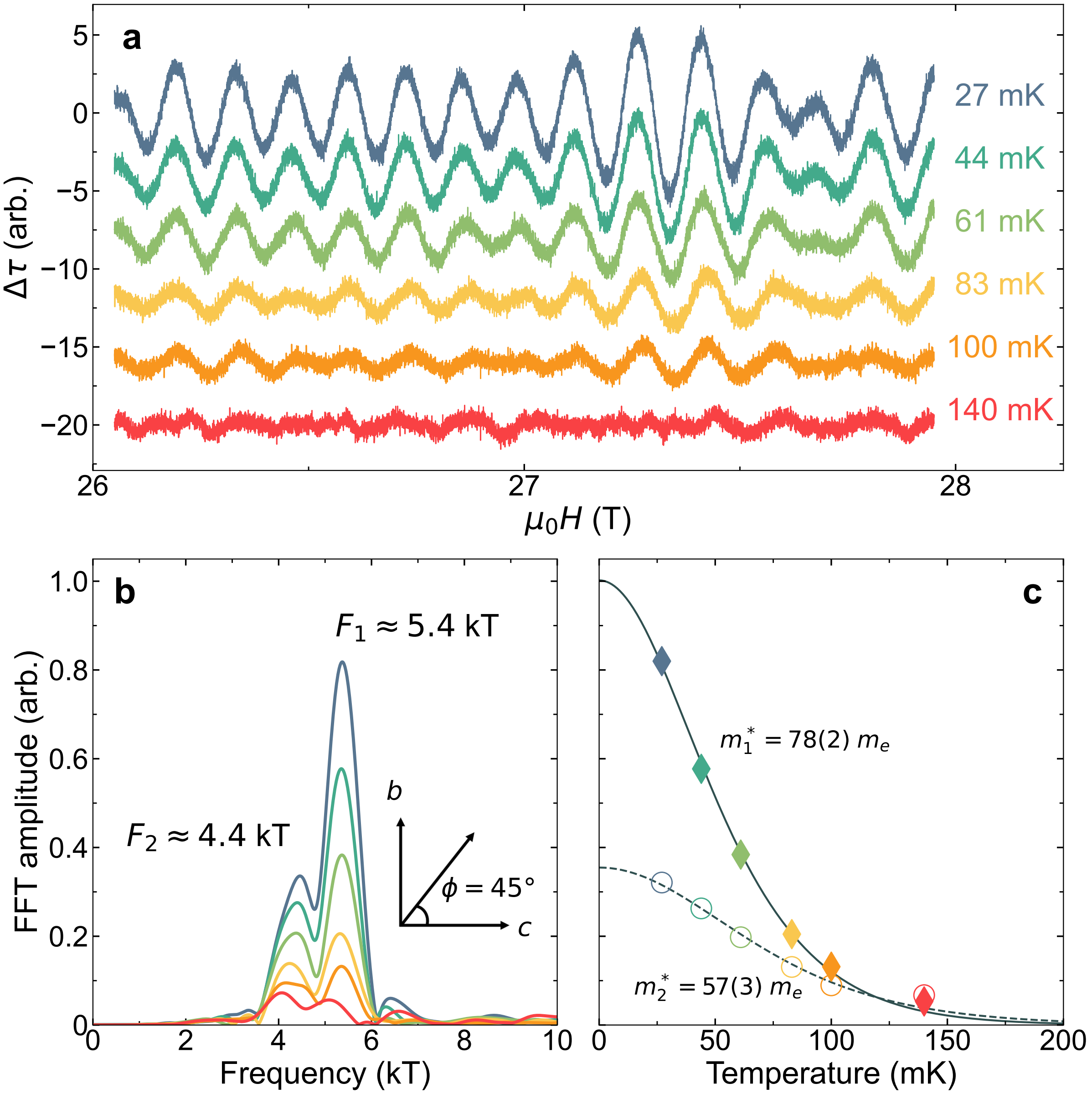


**Fig. S19. Heavy quasiparticle effective masses.** **a**, Oscillatory component of magnetic torque at $\phi = 45°$ from $\vec{c}$ towards $\vec{b}$ over the field range 26-28 T, at various temperatures between 27 mK and 140 mK as indicated. **b**, Fast Fourier transforms (FFTs) of the data in (a). Two prominent frequency branches are observed, at 5.4 kT and 4.4 kT, respectively. **c**, FFT amplitudes from (b) plotted versus temperature. Lines are fits to the Lifshitz-Kosevich theory of temperature damping for magnetic quantum oscillations[34] (see Methods); these fits yield cyclotron effective masses of 78(2) $m_e$ for the 5.4 kT branch, and 57(3) $m_e$ for the 4.4 kT branch; $m_e$ denotes the bare electron mass.

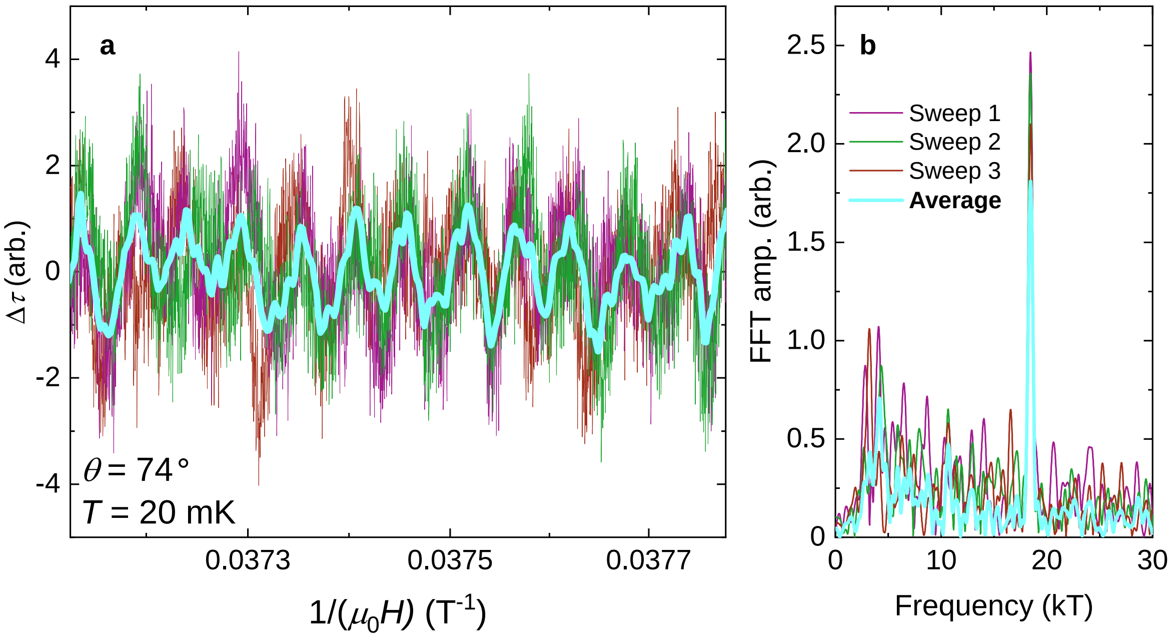


**Fig. S20. Measurements of the high frequency, small amplitude dHvA signal at** $\theta = $ **74°. a**, $\Delta\tau$ from three successive magnetic field sweeps for $\vec{H}$ tilted 74° from $\vec{c}$ towards $\vec{a}$, plotted linearly in inverse field. To maximise the ratio of signal-to-noise, the magnetic field was swept slowly at a rate of 0.05 T/min for each curve. The cyan curve is the averaged, smoothed waveform. **b**, The corresponding FFTs of the data in (a). All curves show a clear peak at 18.5 kT on top of background noise. It is the average of these three individual sweeps that is plotted in Fig. 2 of the main text.

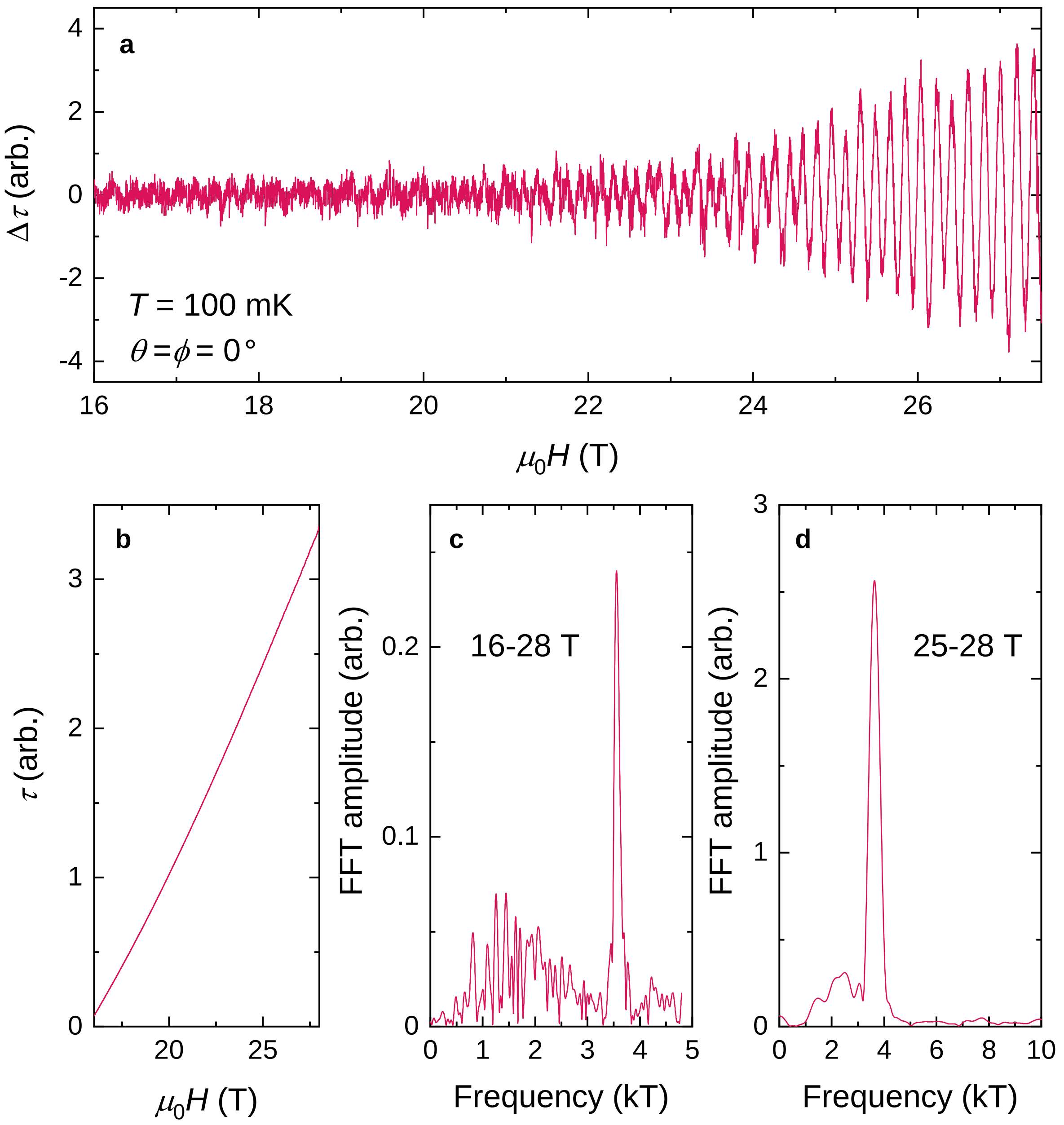


**Fig. S21. Long field sweep for field oriented along the $\vec{c}$ direction. a**, $\Delta\tau$ at 100 mK. Note that the signal to noise of the data presented in this figure is notably poorer than that of the data presented in Fig. 3 of the main text. This is due to the data here being obtained at a considerably faster sweep rate of 0.5 T/min, compared to 0.05 T/min for the $\Delta\tau$ curves in Fig. 3. **b**, Raw torque signal. **c** FFT of the data in (a) over the field interval of 16 T $\leq \mu_0 H \leq$ 28 T, and **d**, over 25 T $\leq \mu_0 H \leq$ 28 T. No slower frequency components are resolved over this wide field range.

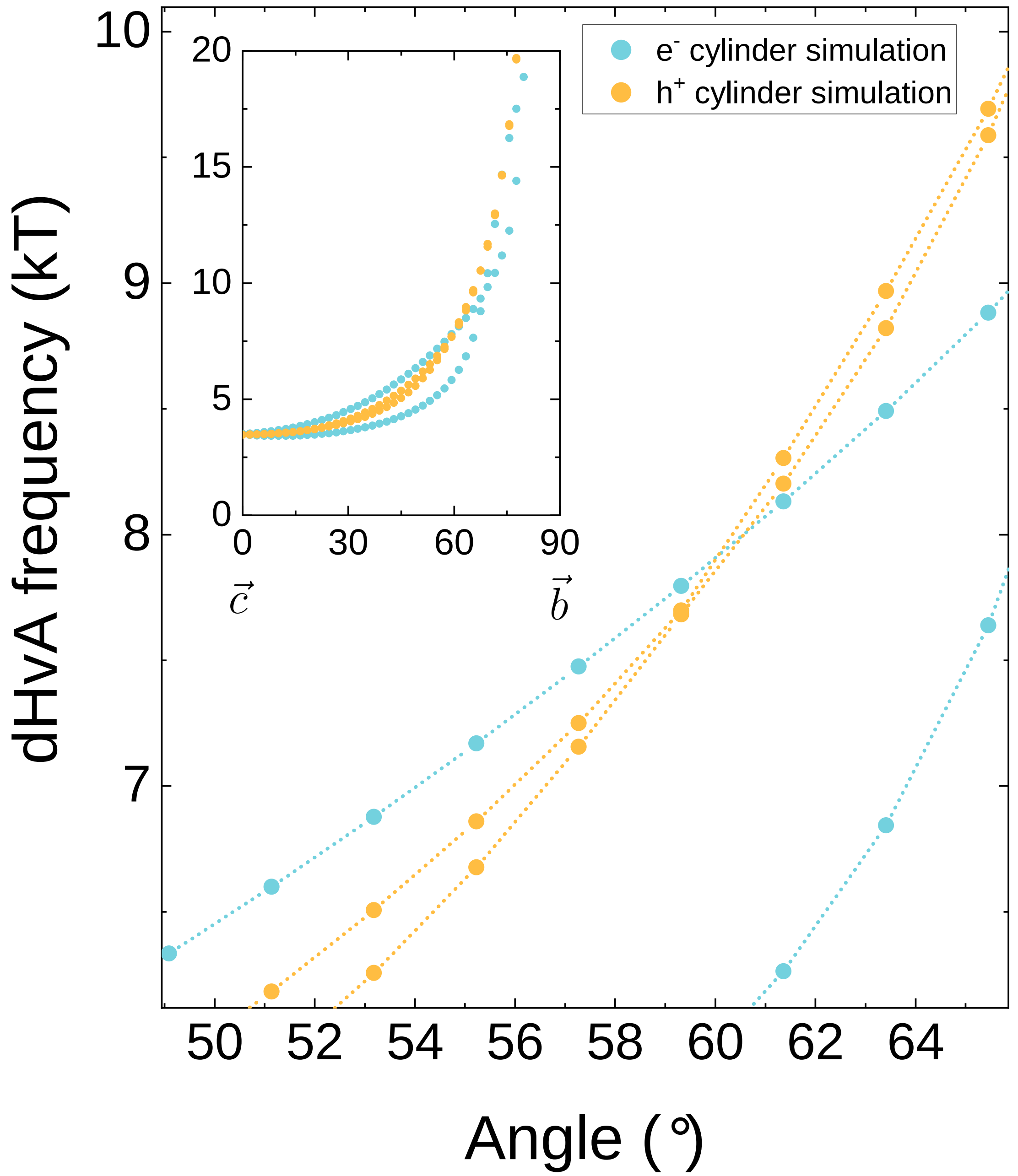


**Fig. S22. Prediction by our Fermi surface simulation of a Yamaji angle coinciding with the orientation of very high magnetic field re-entrant superconductivity.** It has previously been reported that for magnetic fields in excess of 40 T applied in a narrow angular range tilted approximately 60° from the $\vec{c}$ direction towards the $\vec{b}$ direction, re-entrant superconductivity is observed up to at least 70 T (refs.[37,38]). Interestingly, we note that our Fermi surface simulation predicts an intersection of three frequency branches (a crossing of the maxima and minima of the hole sheet intersecting the maxima of the electron sheet) to occur in close proximity to this angle at which the re-entrant superconducting phase is most pronounced.[38] The lines in the main panel are given as a guide to the eye; the inset is the same simulation as plotted in Fig. 4 of the main text.

# References


1. Blaha, P. *et al.* WIEN2k: An APW+lo program for calculating the properties of solids. *J. Chem. Phys.* **152**, 074101 (2020).

2. Ishizuka, J., Sumita, S., Daido, A. & Yanase, Y. Insulator-Metal Transition and Topological Superconductivity in $UTe_2$ from a First-Principles Calculation. *Phys. Rev. Lett.* **123**, 217001 (2019).

3. Aoki, D. *et al.* First Observation of the de Haas–van Alphen Effect and Fermi Surfaces in the Unconventional Superconductor $UTe_2$. *J. Phys. Soc. Jpn.* **91**, 083704 (2022).

4. Aoki, D. *et al.* Unconventional superconductivity in $UTe_2$. *J. Phys. Condens. Matter* **34**, 243002 (2022).

5. Sullivan, C. B. & Kaszynski, A. PyVista: 3D plotting and mesh analysis through a streamlined interface for the Visualization Toolkit (VTK). *J. Open Source Softw.* **4**, 1450 (2019).

6. Weinberger, T. py_FS, GitHub repository [https://github.com/TheoWeinberger/py_FS] (2023).

7. Rourke, P. & Julian, S. Numerical extraction of de Haas–van Alphen frequencies from calculated band energies. *Comput. Phys. Commun.* **183**, 324–332 (2012).

8. Bergemann, C., Mackenzie, A. P., Julian, S. R., Forsythe, D. & Ohmichi, E. Quasi-two-dimensional Fermi liquid properties of the unconventional superconductor $Sr_2RuO_4$. *Adv. Phys.* **52**, 639–725 (2003).

9. Baglo, J. *et al.* Fermi Surface and Mass Renormalization in the Iron-Based Superconductor $YFe_2Ge_2$. *Phys. Rev. Lett.* **129**, 046402 (2022).

10. Hutanu, V. *et al.* Low-temperature crystal structure of the unconventional spin-triplet superconductor $UTe_2$ from single-crystal neutron diffraction. *Acta Crystallogr. B* **76**, 137–143 (2020).

11. Onsager, L. Interpretation of the de Haas-van Alphen effect. *Philos. Mag.* **43**, 1006–1008 (1952).

12. Xu, Y., Sheng, Y. & Yang, Y.-f. Quasi-Two-Dimensional Fermi Surfaces and Unitary Spin-Triplet Pairing in the Heavy Fermion Superconductor $UTe_2$. *Phys. Rev. Lett.* **123**, 217002 (2019).

13. Shim, J. H., Haule, K. & Kotliar, G. X-ray absorption branching ratio in actinides: LDA+DMFT approach. *EPL* **85**, 17007 (2009).

14. Yin, Q. *et al.* Electronic correlation and transport properties of nuclear fuel materials. *Phys. Rev. B* **84**, 195111 (2011).

15. Altarawneh, M. M., Mielke, C. H. & Brooks, J. S. Proximity detector circuits: An alternative to tunnel diode oscillators for contactless measurements in pulsed magnetic field environments. *Rev. Sci. Inst.* **80**, 066104 (2009).

16. Liu, H. *et al.* $f$-electron hybridised Fermi surface in magnetic field-induced metallic $YbB_{12}$. *npj Quantum Mater.* **7**, 12 (2022).

17. Semeniuk, K. *et al.* Truncated mass divergence in a Mott metal (2022). arXiv:2202.04024.

18. Aoki, D. *et al.* de Haas-van Alphen Oscillations for the Field Along c-axis in $UTe_2$ (2023). arXiv:2304.07678.

19. Ashcroft, N. W. & Mermin, D. N. *Solid State Physics* (Harcourt College Publishers, San Diego, CA, 1976).

20. Niu, Q. *et al.* Evidence of Fermi surface reconstruction at the metamagnetic transition of the strongly correlated superconductor $UTe_2$. *Phys. Rev. Res.* **2**, 033179 (2020).

21. Eo, Y. S. *et al.* $c$-axis transport in $UTe_2$: Evidence of three-dimensional conductivity component. *Phys. Rev. B* **106**, L060505 (2022).

22. Kitazawa, K. *et al.* Electronic properties of cuprate superconductors. *Physica C Supcond.* **153-155**, 9–14 (1988).

23. Maeno, Y. *et al.* Superconductivity in a layered perovskite without copper. *Nature* **372**, 532–534 (1994).

24. Hussey, N. E. *et al.* Normal-state magnetoresistance of $Sr_2RuO_4$. *Phys. Rev. B* **57**, 5505–5511 (1998).

25. Scheidemantel, T. J., Ambrosch-Draxl, C., Thonhauser, T., Badding, J. V. & Sofo, J. O. Transport coefficients from first-principles calculations. *Phys. Rev. B* **68**, 125210 (2003).

26. Ziman, J. M. *Principles of the Theory of Solids* (Cambridge University Press, 1972).

27. Nevidomskyy, A. H. Stability of a Nonunitary Triplet Pairing on the Border of Magnetism in $UTe_2$ (2020). arXiv:2001.02699.

28. Harrison, N. & McDonald, R. D. Determining the in-plane Fermi surface topology in high $T_c$ superconductors using angle-dependent magnetic quantum oscillations. *J. Phys.: Condens. Matter* **21**, 192201 (2009).

29. Sebastian, S. E. *et al.* Compensated electron and hole pockets in an underdoped high-$T_c$ superconductor. *Phys. Rev. B* **81**, 214524 (2010).

30. Sebastian, S. E. *et al.* Normal-state nodal electronic structure in underdoped high-Tc copper oxides. *Nature* **511**, 61–64 (2014).

31. Sebastian, S. E. & Proust, C. Quantum oscillations in hole-doped cuprates. *Annu. Rev. Condens. Matter Phys.* **6**, 411–430 (2015).

32. Dessau, D. S. *et al.* Key features in the measured band structure of $Bi_2Sr_2CaCu_2O_{8+\delta}$: Flat bands at $E_F$ and Fermi surface nesting. *Phys. Rev. Lett.* **71**, 2781–2784 (1993).

33. Johannes, M. D. & Mazin, I. I. Fermi surface nesting and the origin of charge density waves in metals. *Phys. Rev. B* **77**, 165135 (2008).

34. Shoenberg, D. *Magnetic Oscillations in Metals* (Cambridge University Press, Cambridge, UK, Cambridge, UK, 1984).

35. Champel, T. & Mineev, V. P. de Haas–van Alphen effect in two- and quasi-two-dimensional metals and superconductors. *Philos. Mag. B* **81**, 55–74 (2001).

36. Dingle, R. B. Some magnetic properties of metals II. The influence of collisions on the magnetic behaviour of large systems. *Proc. R. Soc. Lond. A* **211**, 517–525 (1952).

37. Ran, S. *et al.* Extreme magnetic field-boosted superconductivity. *Nat. Phys.* **15**, 1250–1254 (2019).

38. Helm, T. *et al.* Suppressed magnetic scattering sets conditions for the emergence of 40 T high-field superconductivity in $UTe_2$ (2022). arXiv:2207.08261.
